# Subcutaneous versus transvenous implantable cardioverter-defibrillator therapy: an updated systematic review and meta-analysis

**DOI:** 10.1136/openhrt-2026-004443

**Published:** 2026-10-20

**Authors:** Fahad Muhammad, Eyad Jamileh, Omar Farooq, Mohammad Abusheikha, Kaveh Hosseini, Amr Abdin, G André Ng, Ibrahim Antoun

**Affiliations:** 1 Faculty of Medicine and Dentistry, Queen Mary University of London, London, UK; 2 Department of Medicine, Royal Blackburn Teaching Hospital, East Lancashire Hospitals NHS Trust, Blackburn, UK; 3 School of Medicine, Dentistry and Biomedical Sciences, Queen’s University Belfast, Belfast, UK; 4 Department of Medicine, Blackpool Victoria Hospital, Blackpool, Lancashire, UK; 5 Department of Cardiology, Copenhagen University Hospital Department of Cardiology, Copenhagen, Denmark; 6 Saarland University Hospital Internal Medicine III - Cardiology Angiology and Intensive Care Medicine, Homburg, Germany; 7 Division of Cardiovascular Sciences Clinical Science Wing, University of Leicester, Leicester, UK; 8 National Institute for Health Research, Leicester Biomedical Research Centre, Leicester, UK; 9 Leicester British Heart Foundation Centre of Research Excellence, Leicester, UK; 10 Department of Cardiology, University Hospitals of Leicester NHS Trust, Leicester, UK

**Keywords:** Meta-Analysis, Defibrillators, Implantable, Systematic Reviews as Topic

## Abstract

**Background:**

The subcutaneous implantable cardioverter-defibrillator (S-ICD) avoids intravascular leads but does not provide chronic bradycardia pacing or antitachycardia pacing. We performed an updated systematic review and meta-analysis comparing S-ICD with transvenous ICD therapy, prioritising contemporary randomised evidence and clinically relevant device trade-offs.

**Methods:**

Following Preferred Reporting Items for Systematic Reviews and Meta-Analyses (PRISMA) 2020 (PROSPERO: CRD420261328562), PubMed/MEDLINE, Embase and CENTRAL were searched from inception and rerun on 14 September 2026. Randomised evidence was prioritised, with non-randomised evidence analysed separately as supportive. Published time-to-event HRs were retained where available; sparse binary outcomes were analysed without blanket continuity correction, and incompatible endpoints or follow-up horizons were reported separately.

**Results:**

23 reports representing 15 study/cohort families were included. In the published Prospective Randomized Comparison of Subcutaneous and Transvenous Implantable Cardioverter Defibrillator Therapy (PRAETORIAN)–Avoid Transvenous Leads in Appropriate Subjects (ATLAS) individual-participant-data analysis Transvenous Leads in Appropriate Subjects (ATLAS) individual-participant-data analysis, S-ICD was associated with a higher hazard of first inappropriate shock (HR 1.61, 95% CI 1.06 to 2.45). Trial-level analysis also showed a higher hazard of first appropriate shock (HR 1.54, 95% CI 1.14 to 2.09), although this finding was sensitive to the uncertainty method. Conversely, randomised evidence showed fewer lead-related complications with S-ICD in PRAETORIAN (HR 0.24, 95% CI 0.10 to 0.54) and fewer major lead-related perioperative complications in ATLAS (risk difference −4.4 percentage points, 95% CI −6.9 to −1.9). The two-trial all-cause mortality estimate was imprecise (HR 1.22, 95% CI 0.89 to 1.66). Infection, pneumothorax and tamponade outcomes were sparse and definition-dependent. PRAETORIAN-Extended Long-Term Follow-Up (XL) provided supportive extended follow-up but was not treated as an independent trial.

**Conclusions:**

Randomised evidence suggests fewer lead-related and invasive device complications with S-ICD but a higher hazard of first inappropriate shock. First-appropriate-shock inference was method-sensitive, while mortality and rare-event comparisons remained uncertain. Device selection should therefore consider pacing and antitachycardia-pacing requirements, future procedures, infection severity and patient preferences without inferring equivalence from non-significant results.

**PROSPERO registration number:**

CRD420261328562.

**WHAT IS ALREADY KNOWN ON THIS TOPIC**

- Previous comparative studies and meta-analyses have consistently suggested fewer transvenous lead-related complications with subcutaneous implantable cardioverter-defibrillator (S-ICD) while earlier pooled analyses did not identify a statistically clear difference in inappropriate shocks. However, those syntheses preceded or incompletely incorporated Avoid Transvenous Leads in Appropriate Subjects (ATLAS), long-term Prospective Randomized Comparison of Subcutaneous and Transvenous Implantable Cardioverter Defibrillator Therapy (PRAETORIAN) follow-up and the PRAETORIAN–ATLAS individual-participant-data analysis, leaving uncertainty regarding contemporary shock risk and longer-term device trade-offs.

**WHAT THIS STUDY ADDS**

- This update prioritises randomised evidence, links repeated publications to their parent cohorts and preserves source-defined time-to-event and competing-risk estimands. Contemporary randomised evidence confirms fewer lead-related and invasive device complications with S-ICD but also identifies a higher hazard of first inappropriate shock in the PRAETORIAN–ATLAS Individual Participant Data analysis. Mortality, infection and rare procedural outcomes remain uncertain, and extended PRAETORIAN-Extended Long-Term Follow-Up (XL) findings are interpreted separately from the parent trial.

**HOW THIS STUDY MIGHT AFFECT RESEARCH, PRACTICE OR POLICY**

- These findings emphasise the trade-off between avoiding intravascular lead complications and retaining pacing and antitachycardia-pacing capabilities while limiting inappropriate shocks. Device selection should incorporate anticipated pacing needs, arrhythmia substrate, future upgrade burden, infection severity and patient preferences. Further comparative research should clarify long-term outcomes, patient-reported effects and whether specific clinical subgroups experience different relative benefits or harms.

## Introduction

 Implantable cardioverter-defibrillators (ICDs) remain the cornerstone of therapy for the prevention of sudden cardiac death in patients at risk of life-threatening ventricular arrhythmias.^1^ For decades, transvenous ICDs (TV-ICDs) have been the established standard of care, providing reliable defibrillation, bradycardia pacing and antitachycardia pacing (ATP).^1 2^ However, the long-term utility of TV-ICDs is frequently limited by lead-related complications, including vascular injury, systemic infection and lead fracture or displacement.^3^ These issues often necessitate complex lead extraction procedures, which carry significant perioperative morbidity and mortality.^4^

The subcutaneous ICD (S-ICD) was developed to mitigate these intravascular risks by using an entirely extravascular lead configuration.^5^ By avoiding the heart and central vasculature, this configuration is designed to reduce complications attributable to intravascular leads, including vascular injury, venous obstruction and systemic infection.^5 6^ Unlike TV-ICDs, however, S-ICDs do not provide chronic bradycardia pacing or ATP, creating a distinct clinical trade-off between avoiding transvenous lead complications and retaining pacing and antitachycardia capabilities.^1 5^

The contemporary evidence base has expanded from the Prospective Randomized Comparison of Subcutaneous and Transvenous Implantable Cardioverter Defibrillator Therapy (PRAETORIAN) trial, which demonstrated the non-inferiority of S-ICD compared with TV-ICD,^7^ through its extended follow-up in PRAETORIAN-Extended Long-Term Follow-Up (XL), which extended follow-up of the randomised PRAETORIAN cohort to approximately 8 years,^8^ and the independent Avoid Transvenous Leads in Appropriate Subjects (ATLAS) trial, which provided randomised evidence on perioperative lead-related safety.^9^ Previous meta-analyses by Rordorf *et al*, Fong *et al* and Nso *et al* had already reported fewer lead-related complications with S-ICD and no statistically clear overall difference in inappropriate shocks, although study selection, endpoint definitions and analytical methods differed.^10–12^ More recently, a harmonised individual participant data meta-analysis of PRAETORIAN and ATLAS reported a higher hazard of first inappropriate shock with S-ICD (HR 1.61, 95% CI 1.06 to 2.45).^13^ Accordingly, the principal contribution of the present update is not to re-establish the previously recognised lead-safety signal, but to reassess the balance of long-term lead-related outcomes, first-shock outcomes and clinically relevant device trade-offs using this newer randomised and extended follow-up evidence.

Accordingly, this systematic review and meta-analysis aimed to update the comparative safety and clinical performance of S-ICD versus TV-ICD, prioritising randomised evidence for clinical interpretation and treating non-randomised evidence as supportive. We specifically evaluated lead-related and device-related complications, appropriate and inappropriate shocks and their mechanisms, mortality, infection severity, reintervention, subsequent pacing or upgrade requirements, and patient-reported outcomes, with emphasis on clinically relevant trade-offs rather than equivalence inferred from non-significant findings.

## Methods

### Study design

This review followed Preferred Reporting Items for Systematic Reviews and Meta-Analyses (PRISMA) 2020 and was prospectively registered with PROSPERO (CRD420261328562).^14 15^ The search strategy, population and screening interpretation, risk-of-bias assessment and statistical synthesis were revised following peer review, as described below. The PRISMA checklist is provided in the online supplemental material 1. Ethical approval was not required because only published data were analysed.

### Eligibility criteria

We included randomised controlled trials (RCTs) and non-randomised comparative studies of adults (≥18 years) with a Class I or IIa indication for primary or secondary prevention ICD implantation, directly comparing standalone S-ICD with conventional TV-ICD. The target population had no established requirement for permanent bradycardia pacing, cardiac resynchronisation therapy (CRT) or ATP for recurrent monomorphic ventricular tachycardia. Formal morphological ECG screening was recorded as reported by each study and was not assumed for observational TV-ICD comparators. Mixed or reimplantation populations retained from the original review were treated as source-specific supportive evidence and are identified in online supplemental table S3.

We excluded paediatric-only cohorts, single-arm studies, case reports or series, editorials, reviews, conference abstracts, protocols without outcome data and studies in which standalone S-ICD and TV-ICD outcomes could not be distinguished.

### Data source and search strategy

PubMed/MEDLINE, Embase and CENTRAL were searched from inception through March 2026 and rerun from inception on 14 September 2026. Updated searches used device-specific S-ICD and TV-ICD terms without disease or study design filters. Reference lists and citation searches of eligible reports and relevant reviews were also checked. Complete database-specific strategies are provided in the online supplemental material 1.

### Study selection

Two reviewers (FM and EJ) independently screened records and full texts. Disagreements were resolved by consensus or, when necessary, adjudication by a third reviewer (MA).

### Data extraction

Two reviewers independently extracted study characteristics, setting, analysis population, comparators, follow-up, baseline characteristics, endpoint definitions and effect estimates using a standardised form. The registered main outcomes were device-related complications requiring invasive intervention or revision, lead-related complications and all-cause mortality; additional registered outcomes included tricuspid regurgitation/right-ventricular measures, quality of life and heart-failure hospitalisation.^15^ Shock outcomes and mechanisms, procedural and pocket complications, infection severity, pacing/upgrades and reintervention were treated as additional outcomes.

For each result, we retained the source-defined endpoint, follow-up, analysis population and adjusted or matched effect where available. Patients, recurrent events, shocks and procedures were not interchanged. Publications were linked to their parent cohorts, and at most one overlapping estimate contributed to a given synthesis. PRAETORIAN-XL was treated as extended follow-up of PRAETORIAN, and the PRAETORIAN-ATLAS Individual Participant Data (IPD) analysis was not counted as a third trial.^7–9 13^ Cohort linkage and participant accounting are detailed in online supplemental table S3.

### Quality assessment

Two reviewers independently assessed risk of bias (RoB) at result level using RoB 2 for RCTs and Risk Of Bias In Non-randomised Studies-of Interventions (ROBINS-I) for non-randomised studies, with disagreements resolved by consensus.^16 17^ RoB 2 assessments targeted the effect of assignment to the randomised intervention and used the standard domain-specific signalling questions for each result. For ROBINS-I, the target comparison was initial S-ICD versus TV-ICD strategy with usual subsequent care; important confounders included age, cardiac aetiology, left ventricular ejection fraction, prevention indication, renal disease, previous device or infection, implant era and device programming. Certainty was assessed separately for randomised, extended-follow-up and non-randomised evidence using Grading of Recommendations Assessment, Development and Evaluation (GRADE), considering RoB, inconsistency, indirectness, imprecision and publication bias.^18^ Result-specific judgements and evidence profiles are provided in the online supplemental material 1.

### Data synthesis

Randomised evidence was prioritised for clinical interpretation, with non-randomised evidence analysed separately as supportive evidence; no overall treatment effect was pooled across study designs. Outcomes were synthesised only when definitions, populations, follow-up and effect measures were sufficiently compatible. Published time-to-event HRs were retained where available, while PRAETORIAN-XL subdistribution HRs (sHRs) were reported separately. The published PRAETORIAN-ATLAS IPD estimate was the primary randomised synthesis for first inappropriate shock.^13^ Trial-level shock and mortality HRs were pooled using inverse-variance random-effects models with restricted maximum likelihood (REML); Wald CIs were accompanied by modified Hartung-Knapp and common-effect sensitivity analyses.

For compatible sparse binary outcomes, Mantel-Haenszel relative risks (RRs) were calculated without a blanket continuity correction. Double-zero comparisons remained visible but were not forced into finite RRs. Device-related infection was additionally examined using a binomial-logistic random-effects generalised linear mixed model. Pneumothorax used stratified exact conditional tests, while isolated tamponade was reported source-specifically. Clinically incompatible endpoints or horizons, including quality-of-life instruments and pacing/upgrade outcomes, were not pooled.

Exploratory study design interactions for first appropriate and inappropriate shock compared RCT and matched observational HRs using meta-regression, with modified Hartung-Knapp inference and Wald sensitivity. Absolute effects were reported per 1000 using published fixed-horizon risks or clearly labelled descriptive differences. Heterogeneity was described using I^2^ and between-study variance where applicable. Leave-one-out and risk-of-bias restrictions were used as sensitivity analyses. No retained meta-analysis included ≥10 studies; publication bias was therefore assessed qualitatively rather than using funnel plots or Egger testing. P values and CIs were nominal without adjustment for multiplicity, and non-significance was not interpreted as equivalence.

Statistical analyses were performed in R V.4.5.3 (R Foundation for Statistical Computing, Vienna, Austria) using the meta (V.8.5–0) and metafor (V.5.0–1) packages. Figures were generated in R using custom plotting code.

## Results

### Study selection and characteristics

The updated search identified 990 records. After removal of 579 duplicates, 411 records were screened and 58 reports assessed in full text; 23 reports representing 15 study/cohort families were included (figure 1). Two families were randomised trials and five study comparisons contributed to at least one retained meta-analysis.

**Figure 1:**
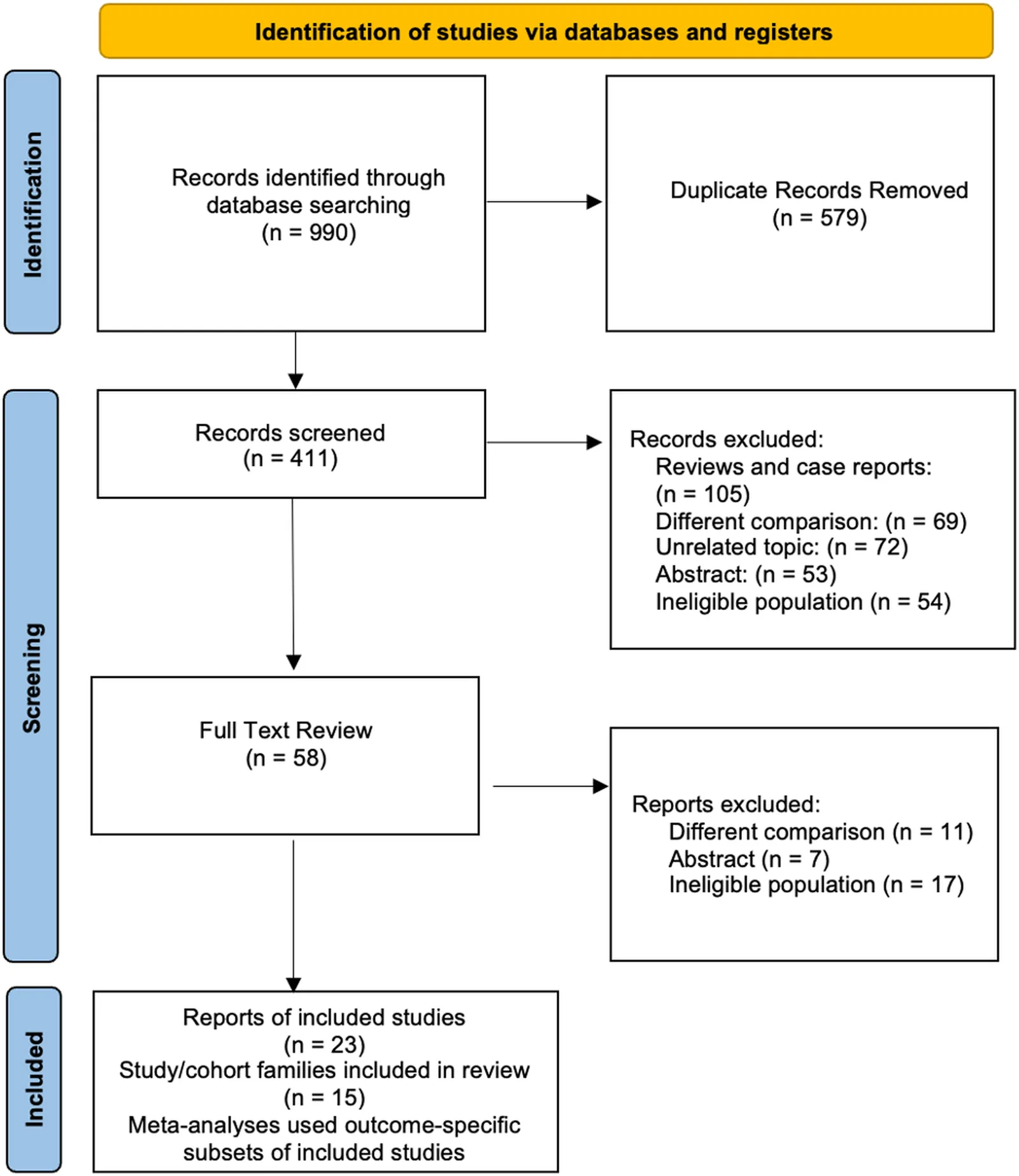
PRISMA flow diagram of study identification and selection. PRISMA 2020 flow diagram summarising identification, screening, eligibility assessment and inclusion of reports in the updated systematic review. The updated search identified 990 records across PubMed/MEDLINE, Embase and CENTRAL. After removal of 579 duplicates, 411 records underwent title and abstract screening, 58 reports were assessed in full text and 23 reports representing 15 study/cohort families were included. Meta-analyses used outcome-specific subsets of eligible studies and linked publications from the same parent cohort were not treated as independent study populations. PRISMA, Preferred Reporting Items for Systematic Reviews and Meta-Analyses.

For the primary first-shock and mortality time-to-event analyses, the two parent-trial clinical analysis populations comprised 1342 participants (673 S-ICD; 669 TV-ICD), whereas procedural analyses used source-specific safety denominators. Linked reports and possible observational overlap precluded a verified review-wide unique-participant total. Study and participant characteristics are presented in tables 1 and 2; cohort linkage and outcome-specific participant accounting are detailed in online supplemental table S3.

**Table 1:** Baseline characteristics of the included studies

Author	Setting, Country	Study design (inc. retrospective/prospective)	Single or Multicentre	Population	Comparison	Follow-up duration	Total sample analysed	Intervention group (S-ICD)	Comparison group (TV-ICD)
**Randomised trial families**									
Knops *et al*^7^, PRAETORIAN	39 centres, Europe and USA	Prospective randomised controlled trial	Multicentre	Patients with a Class I or IIa indication for ICD therapy without an established requirement for bradycardia pacing, CRT or ATP; S-ICD screening applied according to the trial protocol	S-ICD vs TV-ICD	Median 49.1 months; extended follow-up median ≈87.5 months in PRAETORIAN-XL^8^	849 mITT (876 randomised)	426 mITT (442 randomised)	423 mITT (434 randomised)
Healey *et al*^9^, ATLAS	14 centres, Canada	Prospective randomised controlled trial	Multicentre	Patients requiring an ICD, enriched for younger patients or those with inherited arrhythmia/other factors increasing concern regarding transvenous lead complications; no indication for pacing or CRT	S-ICD vs TV-ICD	6-month perioperative safety; mean ≈2.5 years clinical follow-up	503 randomised; 493 implanted/harmonised clinical population	251 randomised; 247 clinical population	252 randomised; 246 clinical population
**Non-randomised comparative cohorts**									
Lee *et al*^23^	National Taiwan University Hospital, Taipei, Taiwan	Retrospective propensity-score matched cohort	Single-centre	S-ICD and TV-ICD recipients; 1:2 propensity-matched comparison	S-ICD vs TV-ICD	Median 2.49 years vs 2.84 years	39 matched	13	26
Palmisano *et al*^21^, POINTED	Seven centres, Italy	Retrospective propensity-score matched cohort	Multicentre	Consecutive adults undergoing de novo ICD implantation and eligible for either device strategy	S-ICD vs TV-ICD	Median 30.3 vs 31.3 months	338 matched	169	169
Brouwer *et al*^22^, SIMPLE/EFFORTLESS	International SIMPLE and EFFORTLESS cohorts	Retrospective propensity-score matched comparative analysis	Multicentre	Adults receiving de novo ICD implantation without an indication for pacing or CRT	S-ICD vs TV-ICD	2.9 vs 3.3 years; 3-year estimates	782 matched	391	391
Liang *et al*^32^	Mayo Clinic, USA	Retrospective directly matched cohort	Single-centre	Patients meeting guideline indications for ICD implantation without indications for permanent pacing, CRT or ATP	S-ICD vs TV-ICD	Mean 23 vs 24 months	172	86	86
Viani *et al*^33^, Rhythm Detect	12 centres, Italy	Retrospective analysis of a prospective registry	Multicentre	Patients undergoing ICD reimplantation following extraction of a previous transvenous ICD	S-ICD vs TV-ICD reimplantation	Median 17 months overall	229	90	139
Boveda *et al*^34^	Clinique Pasteur, Toulouse, France	Prospective matched cohort	Single-centre	Patients undergoing ICD implantation; mixed initial implantation and reimplantation population	S-ICD vs TV-ICD	Mean 6 months	62	31	31
Mithani *et al*^35^	Cooper University Hospital, Camden, New Jersey, USA	Retrospective matched cohort	Single-centre	Patients undergoing ICD implantation and directly matched between S-ICD and TV-ICD groups	S-ICD vs TV-ICD	180 days	182	91	91
Honarbakhsh *et al*^36^	Single tertiary centre, London, UK	Retrospective propensity-score matched cohort	Single-centre	S-ICD recipients matched to TV-ICD recipients; excluded established pacing/CRT indications, monomorphic VT requiring ATP and advisory transvenous leads	S-ICD vs TV-ICD	Mean 31 vs 32 months	138	69	69
Al-Ghamdi *et al*^37^	King Faisal Specialist Hospital and Research Centre, Riyadh, Saudi Arabia	Retrospective comparative cohort	Single-centre	Comparative ICD cohort; included younger patients and participants with previous devices	S-ICD vs TV-ICD	Mean 710.6 vs 632 days	60	30	30
Köbe *et al*^38^	University Hospital Münster, Germany	Retrospective case-matched questionnaire cohort	Single-centre	S-ICD and TV-ICD recipients able to complete standardised patient-reported outcome questionnaires	S-ICD vs TV-ICD	Mean time since implantation 622.1 vs 941.9 days	84 responders*	42	42
Friedman *et al*^39^	National Cardiovascular Data Registry ICD Registry, USA	Retrospective propensity-score matched registry study	Multicentre	Patients undergoing first ICD implantation without a pacing or CRT indication	S-ICD vs single-chamber and dual-chamber TV-ICD	Index admission	5760†	1920	3840†
Brouwer *et al*^40^	Academic Medical Centre Amsterdam and Leiden University Medical Centre, Netherlands	Retrospective propensity-score matched cohort	Multicentre	Propensity-matched S-ICD and single-chamber or dual-chamber TV-ICD recipients; six S-ICD recipients had concomitant transvenous pacemakers	S-ICD vs TV-ICD	Median 3 vs 5 years; 5-year estimates	280	140	140
Köbe *et al*^41^	Münster, Düsseldorf and Munich, Germany	Prospective case-matched study	Multicentre	Case-matched S-ICD and single-chamber TV-ICD recipients; patients with pacing or ATP indications excluded	S-ICD vs single-chamber TV-ICD	Mean ≈217 days; first follow-up at 4 weeks	138	69	69

Data are shown for study/cohort families rather than individual publications. Linked secondary reports and extended follow-up publications are not counted as additional study populations.

The Brouwer *et al* (2018) SIMPLE/EFFORTLESS matched analysis included 782 participants (391 per group); a larger propensity-stratified analysis of 2387 participants from the same source population was used for the adjusted mechanical lead-related outcome and was not counted as an additional cohort.

* Köbe *et al* (2017) included 42 S-ICD and 42 TV-ICD questionnaire responders from 60 participants invited in each group. Previously reported values of 30/30 represented male participants, not analysis denominators.

† Friedman *et al* (2016) comprised one 5760-participant three-arm matched population: 1920 S-ICD recipients, 1920 single-chamber TV-ICD recipients and 1920 dual-chamber TV-ICD recipients. The same S-ICD group was used in both TV-ICD comparisons.

ATLAS, Avoid Transvenous Leads in Appropriate Subjects; ATP, antitachycardia pacing; CRT, cardiac resynchronisation therapy; ICD, implantable cardioverter-defibrillator; inc., including; mITT, modified intention-to-treat; POINTED, Impact on Patient Outcome and Healthcare Utilization of Cardiac Implantable Electronic Devices Complications Registry; PRAETORIAN-XL, Prospective Randomized Comparison of Subcutaneous and Transvenous Implantable Cardioverter Defibrillator Therapy – Long-Term Follow-Up; S-ICD, subcutaneous implantable cardioverter-defibrillator; TV-ICD, transvenous implantable cardioverter-defibrillator; VT, ventricular tachycardia.

**Table 2:** Baseline characteristics of participants included in each study

Author	Age, years (mean±SD or median (IQR))		Male sex, n (%)		Body mass index, kg/m²		Ejection fraction (mean±SD or median (IQR))		Ischaemic cardiomyopathy, n (%)		Non-ischaemic cardiomyopathy, n (%)	
	S-ICD	TV-ICD	S-ICD	TV-ICD	S-ICD	TV-ICD	S-ICD	TV-ICD	S-ICD	TV-ICD	S-ICD	TV-ICD
Lee *et al*^23^	45.0 (30.0–57.0)	44.5 (32.8–54.0)	11 (84.6)	21 (80.8)	24.81 (22.21–26.79)	24.60 (21.87–28.81)	70.70 (67.30–77.05)	64.05 (40.75–69.38)	0 (0)	8 (30.8)	1 (7.7)	4 (15.4)
Healey *et al*^9^	48±11.9	50±11.1	191 (76.1)	182 (72.2)	NR	NR	45±19	46±18	NR	NR	NR	NR
Palmisano *et al*^21^	55.6±13.0	57.4±15.5	138 (81.7)	127 (75.1)	NR	NR	NR	NR	71 (42.0)	60 (35.5)	NR	NR
Knops *et al*^7^	63 (54–69)	64 (56–70)	337 (79.1)	345 (81.6)	27.0 (24.5–30.5)	27.9 (25.2–31.7)	30 (25–35)	30 (25–35)	289 (67.8)	298 (70.4)	99 (23.2)	98 (23.2)
Liang *et al*^32^	45±16	46±16	59 (68.6)	60 (69.8)	29±6	31±8	51±18	49±20	17 (19.8)	22 (25.6)	NR	NR
Viani *et al*^33^	53±13	60±18	80 (89)	104 (75)	26±4	25±4	44±15	44±15	42 (47)	67 (48)	NR	NR
Brouwer *et al*^22^	54±16	55±13	299 (76.5)	319 (81.6)	28±6	28±5	39.4±17.3	39.8±16.9	187 (47.8)	194 (49.6)	NR	NR
Boveda *et al*^34^	58.7±13.2	61.5±11.2	NR	NR	24.2±3.9	28.7±5.0	32.6±7.4	29.6±5.2	15 (48)	22 (71)	NR	NR
Mithani *et al*^35^	54.93±13.61	56.30±12.71	51 (56.0)	50 (54.9)	31.41±14.56	31.27±9.44	26.79±12.08	27.78±11.66	NR	NR	57 (62.6)	51 (56.0)
Al-Ghamdi *et al*^37^	41.03	50.33	23 (76.7)	24 (80)	NR	NR	30.5	28.17	11 (36.7)	14 (46.7)	10 (33.3)	14 (46.7)
Honarbakhsh *et al*^36^	35±13	40±10	52 (75)	52 (75)	NR	NR	57±15	58±13	6 (9)	5 (7)	NR	NR
Köbe *et al*^38^	44.6±12.5	44.7±12.1	30 (71.4)	30 (71.4)	25.6±4.9	27.1±7.2	49.0±13.7	44.8±16.6	NR	NR	NR	NR
Friedman *et al*^39^	54.0 (15.1)	53.9 (15.1)	1293 (67.3)	2609 (67.9)	29.9±6.8	29.9±7.2	31.2±13.7	31.3±13.9	879 (45.8)	1747 (45.5)	NR	NR
Brouwer *et al*^40^	41 (26–52)	42 (32–50)	84 (60)	87 (62.1)	NR	NR	50	49	26 (19)	41 (29)	28 (20)	30 (21)
Köbe *et al*^41^	45.7±15.7	47.7±14.7	50 (72.5)	50 (72.5)	NR	NR	46.2±15.6	40.6±15.9	NR	NR	NR	NR

Author	Genetic arrhythmia syndrome		Dilated cardiomyopathy, n (%)		Hypertrophic cardiomyopathy, n (%)		Brugada syndrome, n (%)		Idiopathic ventricular fibrillation, n (%)		Congenital heart disease, n (%)		Primary prevention indication, no. (%)	
	S-ICD	TV-ICD	S-ICD	TV-ICD	S-ICD	TV-ICD	S-ICD	TV-ICD	S-ICD	TV-ICD	S-ICD	TV-ICD	S-ICD	TV-ICD
Lee *et al*^23^	7 (53.8)	6 (23.1)	NR	NR	2 (15.4)	4 (15.4)	NR	NR	3 (23.1)	3 (11.5)	0 (0)	1 (3.8)	NR	NR
Healey *et al*^9^	NR	NR	56 (22.3)	60 (23.8)	45 (17.9)	48 (19.0)	5 (2.0)	7 (2.8)	47 (18.7)	37 (14.7)	0 (0.0)	1 (0.4)	NR	NR
Palmisano *et al*^21^	NR	NR	51 (30.2)	56 (33.1)	17 (10.1)	20 (11.8)	NR	NR	7 (4.1)	11 (6.5)	NR	NR	NR	NR
Knops *et al*^7^	20 (4.7)	18 (4.3)	NR	NR	NR	NR	NR	NR	11 (2.6)	5 (1.2)	3 (0.7)	3 (0.7)	NR	NR
Liang *et al*^32^	NR	NR	17 (19.8)	17 (19.8)	32 (37.2)	33 (38.4)	NR	NR	NR		NR	NR	68 (79.1)	68 (79.1)
Viani *et al*^33^	NR	NR	14 (16)	19 (14)	5 (5)	13 (9)	NR	NR	NR	NR	14 (16)	11 (8)	NR	NR
Brouwer *et al*^22^	NR	NR	41 (10.5)	33 (8.4)	37 (9.5)	39 (10.0)	NR	NR	NR	NR	NR	NR	272 (69.6)	279 (71.4)
Boveda *et al*^34^	NR	NR	3 (10)	5 (16)	4 (13)	4 (13)	4 (13)	0 (0)	2 (6)	0 (0)	NR	NR	18 (58)	20 (65)
Mithani *et al*^35^	NR	NR	NR	NR	NR	NR	NR	NR	NR	NR	NR	NR	74 (81.3)	70 (76.9)
Al-Ghamdi *et al*^37^	4 (13.3)	2 (6.7)	NR	NR	NR	NR	NR	NR	NR	NR	5 (16.7)	0 (0.0)	25 (83)	26 (86.7)
Honarbakhsh *et al*^36^	NR	NR	4 (6)	5 (7)	41 (59)	42 (61)	4 (6)	4 (6)	6 (9)	6 (9)	1 (1)	1 (1)	56 (81)	56 (81)
Köbe *et al*^38^	NR	NR	7 (16.7)	12 (28.6)	10 (23.8)	3 (7.1)	NR	NR	10 (23.8)	12 (28.6)	4 (9.5)	5 (11.9)	26 (61.9)	23 (54.8)
Friedman *et al*^39^	NR	NR	846 (44.1)	1677 (43.7)	NR	NR	21 (1.1)	34 (0.9)	17 (0.9)	32 (0.8)	NR	NR	NR	NR
Brouwer *et al*^40^	75 (54)	54 (39)	NR	NR	NR	NR	NR	NR	NR	NR	5 (4)	12 (9)	93 (66)	86 (61)
Köbe *et al*^41^	NR	NR	25 (36.2)	32 (46.4)	10 (14.5)	4 (5.8)	NR	NR	22 (31.9)	18 (26.1)	3 (4.4)	3 (4.4)	41 (59.4)	34 (50.0)

Author	Secondary prevention indication, no. (%)		Hypertension, n (%)		Diabetes, n (%)		Atrial Fibrillation, n (%)		HF: NYHA class I, no. (%)		HF: NYHA class II, no. (%)	
	S-ICD	TV-ICD	S-ICD	TV-ICD	S-ICD	TV-ICD	S-ICD	TV-ICD	S-ICD	TV-ICD	S-ICD	TV-ICD
Lee *et al*^23^	5 (38.5)	11 (42.3)	3 (23.1)	7 (26.9)	0 (0)	2 (7.7)	2 (15.4)	3 (11.5)	12 (92.3)	15 (57.7)	1 (7.7)	9 (34.6)
Healey *et al*^9^	72 (28.7)	65 (25.8)	88 (35)	88 (35)	49 (19.5)	49 (19.4)	NR	NR	NR	NR	NR	NR
Palmisano *et al*^21^	27 (16.0)	39 (23.1)	89 (52.7)	95 (56.2)	31 (18.3)	34 (20.1)	39 (23.1)	50 (29.6)	49 (29.0)	54 (32.0)	69 (40.8)	77 (45.6)
Knops *et al*^7^	80 (18.8)	84 (19.9)	227 (53.5)	240 (57.3)	112 (26.3)	126 (29.9)	115 (27.0)	93 (22.1)	144 (34.0)	134 (31.8)	205 (48.5)	223 (53.0)
Liang *et al*^32^	NR	NR	27 (31.4)	32 (37.2)	7 (8.1)	13 (15.1)	11 (12.8)	12 (14.0)	NR	NR	NR	NR
Viani *et al*^33^	NR	NR	NR	NR	18 (20)	32 (23)	11 (12)	28 (20)	NR	NR	NR	NR
Brouwer *et al*^22^	NR	NR	168 (43.0)	169 (43.2)	66 (16.9)	64 (16.4)	80 (20.5)	77 (19.7)	NR	NR	88 (22.5)	92 (23.5)
Boveda *et al*^34^	NR	NR	NR	NR	NR	NR	NR	NR	NR	NR	NR	NR
Mithani *et al*^35^	NR	NR	74 (81.3)	72 (79.1)	37 (40.7)	38 (41.8)	14 (15.4)	15 (16.5)	14 (15.4)	16 (17.6)	50 (54.9)	47 (51.6)
Al-Ghamdi *et al*^37^	NR	NR	7 (23.3)	16 (53.3)	6 (20)	16 (53.3)	3 (10)	7 (23.3)	16 (53.3)	4 (13.3)	10 (33.3)	20 (66.7)
Honarbakhsh *et al*^36^	13 (19)	13 (19)	6 (9)	4 (6)	0	0	NR	NR	NR	NR	NR	NR
Köbe *et al*^38^	16 (38.1)	19 (45.2)	NR	NR	NR	NR	NR	NR	NR	NR	NR	NR
Friedman *et al*^39^	NR	NR	1371 (71.4)	2748 (71.6)	689 (35.9)	1379 (35.9)	322 (16.8)	693 (18.0)	429 (22.3)	860 (22.4)	913 (47.6)	1818 (47.3)
Brouwer *et al*^40^	NR	NR	30 (21)	34 (24)	8 (6)	5 (4)	13 (9)	21 (15)	103 (74)	102 (73)	30 (21)	31 (22)
Köbe *et al*^41^	28 (40.6)	35 (50.7)	NR	NR	NR	NR	NR	NR	NR	NR	NR	NR

Author	HF: NYHA class III or IV, no. (%)		QRS duration, ms		TIA or ischaemic stroke, n (%)		Myocarditis or prior MI, n (%)		Previous ICD implant, n (%)		Channelopathy, n (%)	
	S-ICD	TV-ICD	S-ICD	TV-ICD	S-ICD	TV-ICD	S-ICD	TV-ICD	S-ICD	TV-ICD	S-ICD	TV-ICD
Lee *et al*^23^	0 (0)	2 (7.7)	NR	NR	0 (0)	2 (7.7)	NR	NR	NR	NR	NR	NR
Healey *et al*^9^	NR	NR	NR	NR	9 (3.6)	9 (3.6)	NR	NR	NR	NR	NR	NR
Palmisano *et al*^21^	51 (30.2)	38 (22.5)	NR	NR	9 (5.3)	6 (3.6)	NR	NR	NR	NR	NR	NR
Knops *et al*^7^	74 (17.5)	64 (15.2)	NR	NR	NR	NR	NR	NR	NR	NR	NR	NR
Liang *et al*^32^	NR	NR	NR	NR	NR	NR	NR	NR	NR	NR	NR	NR
Viani *et al*^33^	9 (10)	17 (12)	109±22	121±26	NR	NR	NR	NR	NR	NR	15 (17)	29 (21)
Brouwer *et al*^22^	45 (11.5)	38 (9.7)	106±22	106±20	30 (7.7)	29 (7.4)	NR	NR	NR	NR	35 (9.0)	34 (8.7)
Boveda *et al*^34^	NR	NR	NR	NR	NR	NR	NR	NR	8 (25.8)	0 (0)	NR	NR
Mithani *et al*^35^	27 (29.7)	28 (30.8)	98.93±16.92	99.23±16.90	13 (14.3)	4 (4.4%)	NR	NR	NR	NR	NR	NR
Al-Ghamdi *et al*^37^	4 (13.3)	6 (20)	NR	NR	NR	NR	10 (33.3)	10 (33.3)	NR	NR	NR	NR
Honarbakhsh *et al*^36^	NR	NR	NR	NR	NR	NR	NR	NR	NR	NR	NR	NR
Köbe *et al*^38^	NR	NR	NR	NR	NR	NR	NR	NR	NR	NR	NR	NR
Friedman *et al*^39^	578 (30.1)	1162 (30.3)	99.8±15.3	99.8±16.1	NR	NR	794 (41.4)	1588 (41.4)	NR	NR	NR	NR
Brouwer *et al*^40^	7 (5)	7 (5)	98 (88–108)	100 (90–113)	NR	NR	33 (24)	38 (27)	NR	NR	NR	NR
Köbe *et al*^41^	NR	NR	NR	NR	NR	NR	NR	NR	NR	NR	NR	NR

PRAETORIAN-XL is extended follow-up of PRAETORIAN and is not shown as an independent baseline population.

HF, heart failure; ICD, implantable cardioverter-defibrillator ; MI, myocardial infarction; NR, not reported; NYHA, New York Heart Association; PRAETORIAN-XL, Prospective Randomized Comparison of Subcutaneous and Transvenous Implantable Cardioverter Defibrillator Therapy – Extended Long-Term Follow-Up; S-ICD, subcutaneous implantable cardioverter-defibrillator; TIA, transient ischaemic attack; TV-ICD, transvenous implantable cardioverter-defibrillator.

### Primary outcomes

#### Device-related and lead-related complications

At 48 months, PRAETORIAN reported fewer device-related complications requiring invasive intervention (HR 0.59, 95% CI 0.35 to 0.99) and lead-related complications (HR 0.24, 95% CI 0.10 to 0.54) with S-ICD.^19^ Descriptive differences in published risks were 40 and 52 fewer per 1000, respectively. ATLAS reported a 6-month major lead-related perioperative complication risk difference of −4.4 percentage points (95% CI −6.9 to −1.9), equivalent to 44 fewer per 1000 (figures 2 and 3).^9^

**Figure 2:**
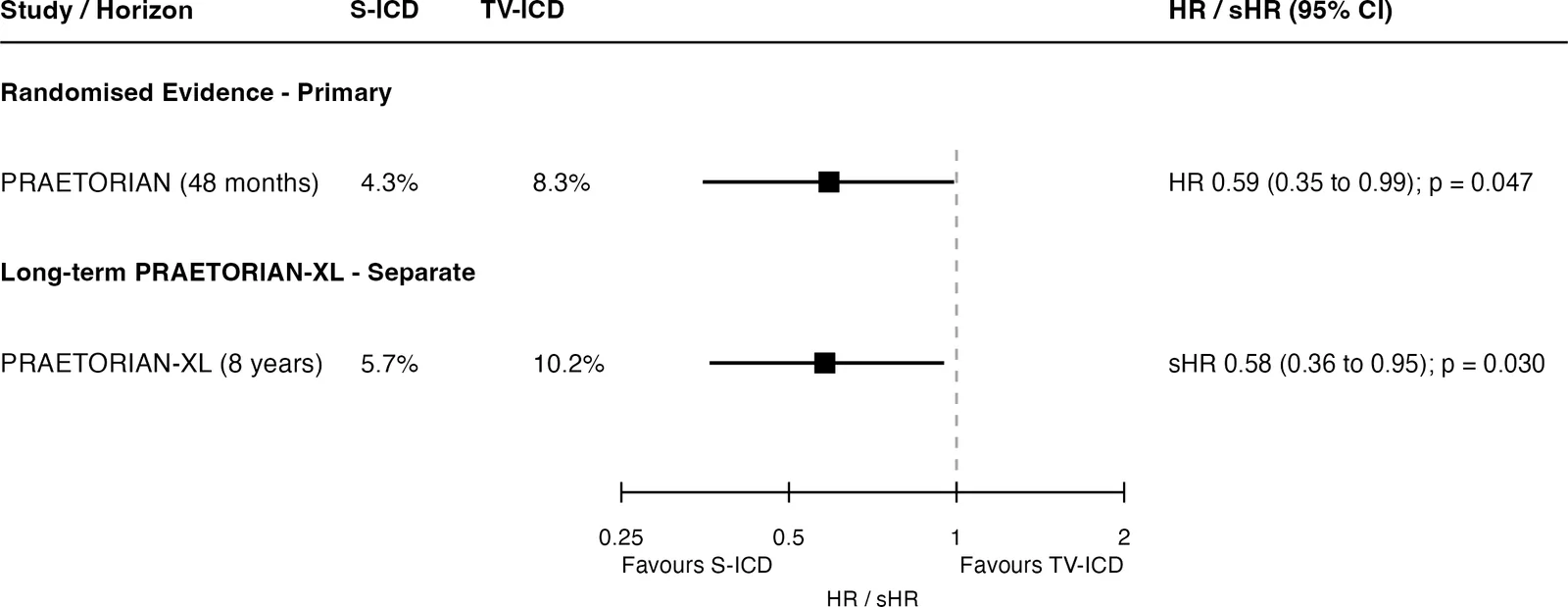
Major and invasive device-related complications with S-ICD versus TV-ICD therapy. Source-specific randomised evidence comparing major or invasive device-related complications with S-ICD versus TV-ICD therapy. PRAETORIAN reports device-related complications requiring invasive intervention at 48 months using a Cox HR, while PRAETORIAN-XL reports long-term major/invasive device complications using a competing-risk sHR. PRAETORIAN-XL represents extended follow-up of the PRAETORIAN cohort and is shown separately rather than pooled with the parent-trial result. Effect estimates below 1 favour S-ICD. Both outcomes are defined by the need for invasive intervention. PRAETORIAN-XL, Prospective Randomized Comparison of Subcutaneous and Transvenous Implantable Cardioverter Defibrillator Therapy - Extended Long-Term Follow-Up; sHR, subdistribution HR; S-ICD, subcutaneous implantable cardioverter-defibrillator; TV-ICD, transvenous implantable cardioverter-defibrillator.

**Figure 3:**
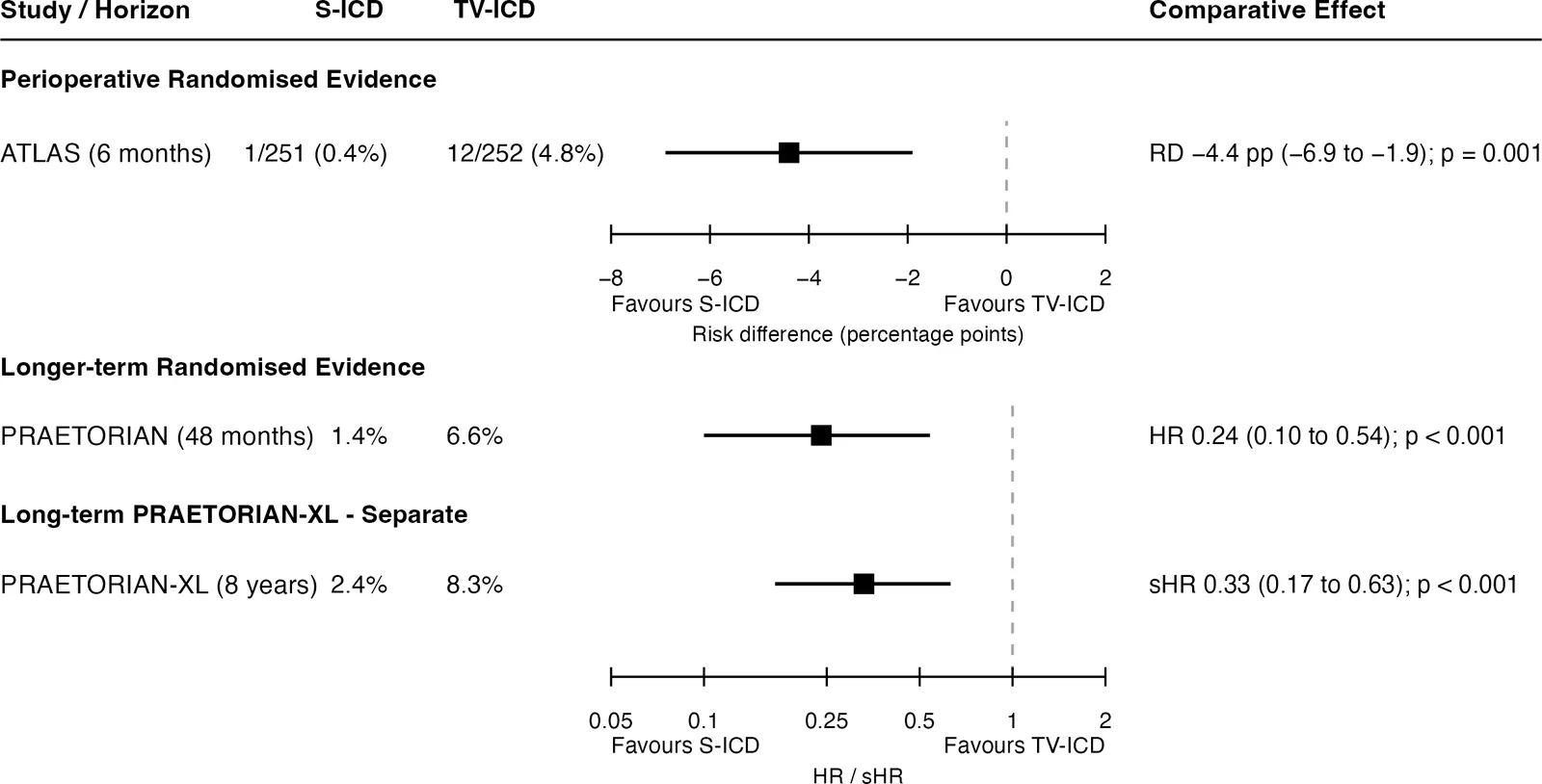
Lead-related complications with S-ICD versus TV-ICD therapy. Source-specific randomised evidence comparing lead-related complications with S-ICD versus TV-ICD therapy. ATLAS reports the 6-month major lead-related perioperative complication risk difference. PRAETORIAN reports lead-related complications at 48 months using a Cox HR, while PRAETORIAN-XL extended follow-up is reported separately using the published competing-risk sHR. These estimates differ in effect measure and follow-up and were therefore not pooled. PRAETORIAN-XL represents extended follow-up of the PRAETORIAN cohort and is not an independent trial. Effect estimates below zero for the ATLAS risk difference and below 1 for the PRAETORIAN HR/sHR favour S-ICD. ATLAS reports a 6-month RD; PRAETORIAN and PRAETORIAN-XL report HR and sHR, respectively. No pooled effect is shown. ATLAS, Avoid Transvenous Leads in Appropriate Subjects; PRAETORIAN**-**XL, Prospective Randomized Comparison of Subcutaneous and Transvenous Implantable Cardioverter Defibrillator Therapy – Extended Long-Term Follow-Up; RD, risk difference; sHR, subdistribution HR; S-ICD, subcutaneous implantable cardioverter-defibrillator; TV-ICD, transvenous implantable cardioverter-defibrillator.

During extended follow-up, PRAETORIAN-XL reported an sHR of 0.58 (95% CI 0.36 to 0.95) for major/invasive device complications and 0.33 (95% CI 0.17 to 0.63) for lead-related complications; descriptive differences in 8-year risks were 45 and 59 fewer per 1000, respectively.^8^ PRAETORIAN-XL was extended follow-up of the parent cohort and was therefore reported separately rather than pooled with the parent-trial estimates.

#### Mortality

The randomised two-trial estimate for all-cause mortality was HR 1.22 (95% CI 0.89 to 1.66; I^2^=0%).^7 9^ Separately, PRAETORIAN-XL reported an 8-year sHR of 1.03 (95% CI 0.81 to 1.32).^8^ Neither estimate established equivalent survival (figure 4).

**Figure 4:**
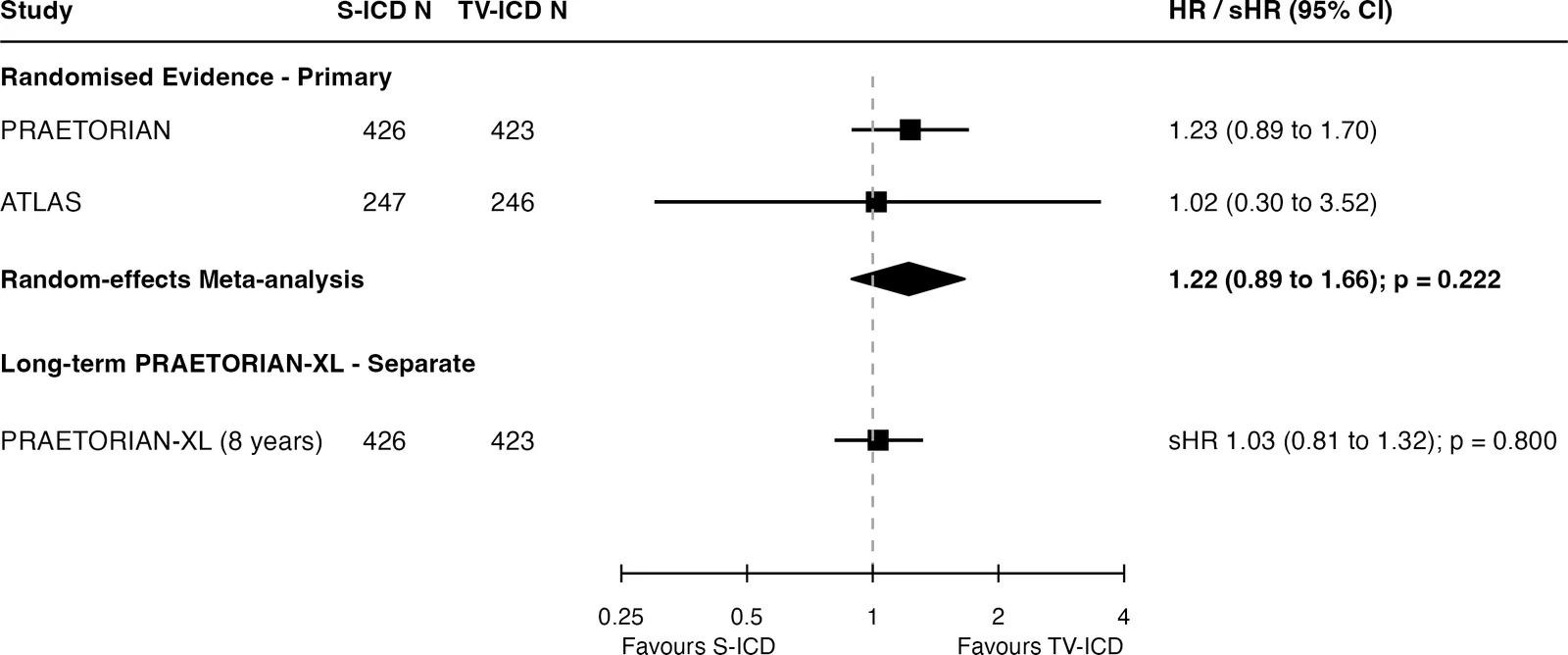
All-cause mortality with S-ICD versus TV-ICD therapy. Forest plot of all-cause mortality with S-ICD versus TV-ICD therapy. The primary parent-trial analysis pools published PRAETORIAN and ATLAS Cox HRs using an inverse-variance random-effects model with restricted maximum likelihood estimation. PRAETORIAN-XL is shown separately because it represents extended follow-up of the PRAETORIAN cohort and reports a competing-risk sHR. Effect estimates are oriented as S-ICD versus TV-ICD. CIs crossing 1 indicate uncertainty and should not be interpreted as demonstrating equivalent survival. Randomised evidence is primary. Other observational mortality data were not pooled. ATLAS, Avoid Transvenous Leads in Appropriate Subjects; PRAETORIAN-XL, Prospective Randomized Comparison of Subcutaneous and Transvenous Implantable Cardioverter Defibrillator Therapy – Extended Long-Term Follow-Up; sHR, subdistribution HR; S-ICD, subcutaneous implantable cardioverter-defibrillator; TV-ICD, transvenous implantable cardioverter-defibrillator.

### Additional outcomes

#### First shocks and inappropriate-shock mechanisms

The published PRAETORIAN-ATLAS IPD analysis found a higher hazard of first inappropriate shock with S-ICD (HR 1.61, 95% CI 1.06 to 2.45; p=0.030).^13^ Four-year risks were 9.6% vs 6.3%, corresponding to a descriptive difference of 33 more first inappropriate shocks per 1000 with S-ICD (figure 5). Trial-level pooling of first appropriate shock yielded HR 1.54 (95% CI 1.14 to 2.09; I^2^=0%; p=0.005)^9 20^; inference was sensitive to the uncertainty method, as reported in the sensitivity analyses (online supplemental figure S1).

**Figure 5:**
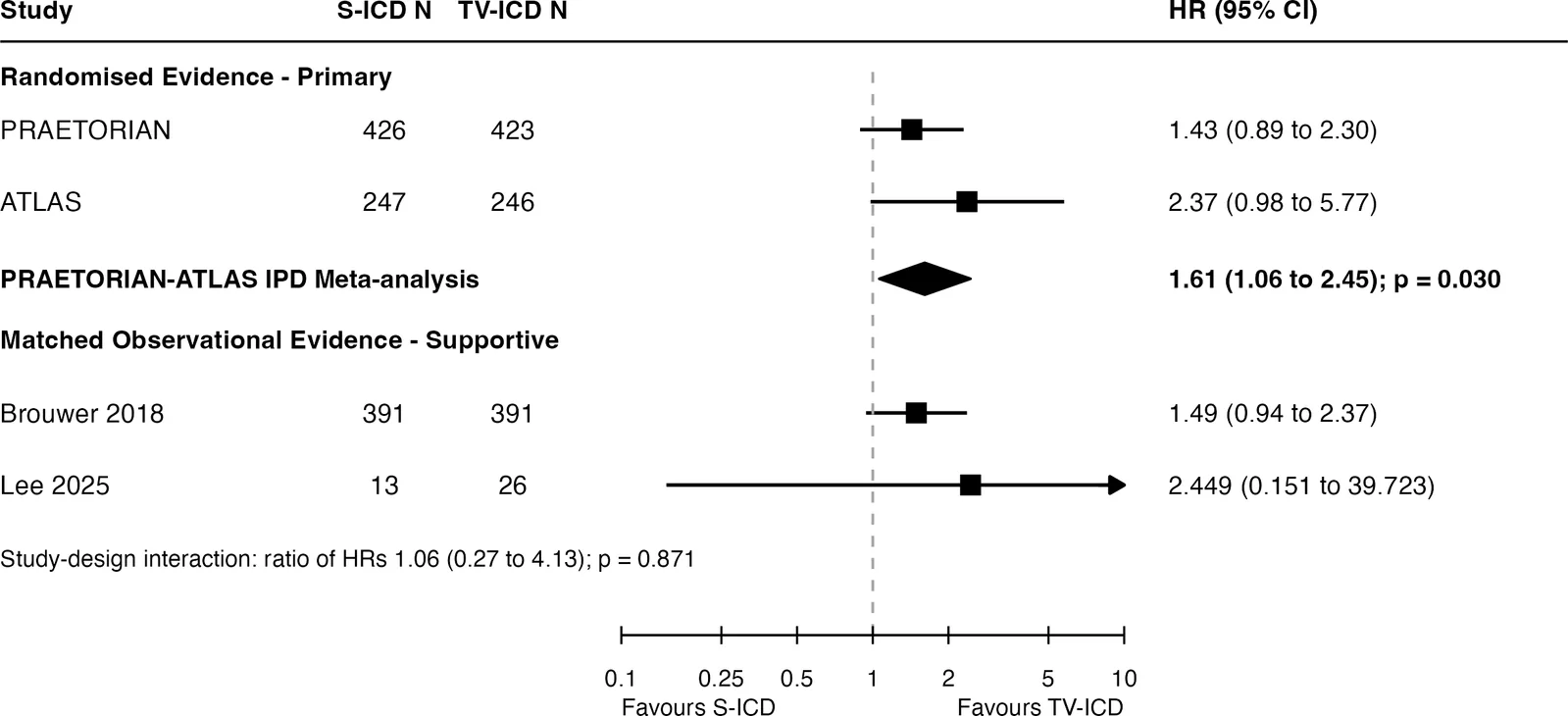
First inappropriate shock with S-ICD versus TV-ICD therapy. Forest plot comparing the hazard of first inappropriate shock with S-ICD versus TV-ICD therapy. Randomised evidence is presented as the primary evidence base, including the published harmonised PRAETORIAN-ATLAS IPD meta-analysis. Trial-level PRAETORIAN and ATLAS estimates are shown for context but were not pooled again with the IPD estimate to avoid double counting. Matched observational estimates are presented separately as supportive evidence. Effect estimates are oriented as S-ICD versus TV-ICD; HRs greater than 1 indicate a higher hazard with S-ICD. The study-design interaction is exploratory and should not be interpreted as evidence of equivalence between randomised and observational effects. Randomised evidence is primary; matched observational evidence is supportive. The pooled estimate is the published PRAETORIAN-ATLAS IPD analysis of the two randomised trials. ATLAS, Avoid Transvenous Leads in Appropriate Subjects; IPD, Individual Participant Data; PRAETORIAN, Prospective Randomized Comparison of Subcutaneous and Transvenous Implantable Cardioverter Defibrillator Therapy; S-ICD, subcutaneous implantable cardioverter-defibrillator; TV-ICD, transvenous implantable cardioverter-defibrillator.

In the IPD analysis, the mechanisms of inappropriate shock differed between device strategies: cardiac oversensing (HR 15.07, 95% CI 3.60 to 63.15) and electromagnetic interference/myopotentials (HR 8.19, 95% CI 1.88 to 35.64) were more frequent with S-ICD, whereas inappropriate shocks attributed to atrial arrhythmias were less frequent (HR 0.37, 95% CI 0.19 to 0.71).^13^

#### Device-related infection

ATLAS reported device infection requiring surgery at 6 months in 2/251 S-ICD vs 1/252 TV-ICD recipients.^9^ PRAETORIAN reported device infection requiring extraction in 4/426 vs 8/423 patients during parent-trial follow-up, while systemic device-related infection at 48 months occurred in 0/426 vs 5/423 patients, respectively.^19^ These clinically distinct infection endpoints and follow-up periods were reported separately rather than pooled.

In supportive matched observational evidence, the Mantel-Haenszel synthesis of Palmisano, Brouwer and Lee yielded RR 2.06 (95% CI 0.78 to 5.42; p=0.145; n=1159).^21–23^ This estimate remained highly uncertain and did not incorporate within-pair covariance or eliminate residual confounding (online supplemental figure 2).

#### Procedural complications

Within the reported procedural windows, pneumothorax occurred in 0/251 vs 2/252 participants in ATLAS and 0/426 vs 4/423 in PRAETORIAN (exact stratified p=0.031).^9 19^ No pooled RR was estimated (online supplemental figure 3). Isolated cardiac tamponade occurred in 0/251 vs 0/252 participants in ATLAS and 0/426 vs 2/423 in PRAETORIAN; the ATLAS double-zero comparison was retained, and its broader pericardial/perforation composite was not substituted for isolated tamponade^9 19^ (online supplemental figure S4).

#### Pacing, upgrades and device changes

In PRAETORIAN-XL, CRT-D upgrades occurred in 27/426 (6.3%) S-ICD and 32/423 (7.6%) TV-ICD recipients.^7 8^ Device changes for subsequent bradycardia-pacing and ATP indications occurred in 11/426 (2.6%) and 3/426 (0.7%) S-ICD recipients, respectively, compared with none in the TV-ICD group; these architecture-dependent device changes should not be interpreted as direct comparisons of the biological incidence of new pacing indications. ATLAS reported an HR of 0.78 (95% CI 0.38 to 1.61; p=0.500) for ICD/lead revision (online supplemental figure S5).^9^

#### Quality of life

Heterogeneous instruments and assessment times precluded pooling of patient-reported outcomes. In ATLAS, the adjusted 6-month difference in Florida Patient Acceptance Survey (FPAS) device-acceptance score was −0.75 (95% CI −3.15 to 1.66; p=0.510), with adjustment for the 1-month postimplant FPAS score.^24^ ATLAS Short Form-36 (SF-36) physical and mental component estimates and PRAETORIAN 30-month Duke Activity Status Index (DASI) functional-capacity, SF-36 bodily pain and mental health comparisons showed no statistically clear between-device differences^24 25^ (online supplemental figure S6).

### Sensitivity analysis

Modified Hartung-Knapp sensitivity analyses produced substantially wider 95% CIs for the trial-level time-to-event estimates: first inappropriate shock, HR 1.60 (95% CI 0.11 to 24.13); first appropriate shock, HR 1.54 (95% CI 0.22 to 10.87); and all-cause mortality, HR 1.22 (95% CI 0.16 to 9.24). Common-effect/Wald estimates were identical to the corresponding REML/Wald results because the estimated between-study variance was zero. The published IPD analysis remained the primary randomised synthesis for first inappropriate shock.

Exploratory RCT-to-matched-observational ratios of HRs were 1.06 (95% CI 0.27 to 4.13; p=0.871) for first inappropriate shock and 2.25 (95% CI 0.73 to 6.92; p=0.090) for first appropriate shock using modified Hartung-Knapp inference. The latter interaction was method-sensitive, excluding 1 with Wald but not modified Hartung-Knapp inference. Infection estimates remained imprecise across alternative sparse-event models; omission of Brouwer 2018 reversed the direction of the Mantel-Haenszel point estimate (online supplemental table S2).

#### Risk of bias and certainty of evidence

Result-specific RoB 2 assessments identified some concerns for the parent-trial shock and mortality results, while PRAETORIAN-XL extension outcomes were at high RoB. GRADE certainty was low for first-shock and parent-trial lead/invasive-complication outcomes and very low for mortality, infection, rare procedural outcomes, extension outcomes and the assessed observational evidence (online supplemental table S1). Restriction of observational analyses to low/moderate ROBINS-I risk left no eligible shock HR estimates and only one infection/bleeding cohort (online supplemental table S2; online supplemental figures 7–10).

### Publication bias

No retained meta-analysis included ≥10 independent studies. Publication bias was therefore assessed qualitatively rather than using funnel plots or Egger’s regression, and publication bias could not be excluded.

## Discussion

This updated systematic review and meta-analysis provides a contemporary comparison of S-ICD and TV-ICD, incorporating PRAETORIAN long-term follow-up, ATLAS and the published PRAETORIAN-ATLAS IPD analysis.^8 9 13^ Randomised evidence indicated a trade-off: fewer lead-related and invasive device complications with S-ICD, but a higher hazard of first inappropriate shock.^9 13 19^ The trial-level first-appropriate-shock estimate was also higher with S-ICD, although inference was method-sensitive. Survival equivalence was not established. GRADE certainty was low for first-shock and parent-trial lead/invasive-complication outcomes and very low for mortality, rare procedural events and extension outcomes.

Previous meta-analyses by Rordorf *et al*, Fong *et al* and Nso *et al* had already reported fewer lead-related complications with S-ICD without a statistically clear overall difference in inappropriate shocks, although study selection and methods differed.^10–12^ Our lead-related findings are therefore principally confirmatory. ATLAS adds randomised perioperative safety evidence, PRAETORIAN-XL extends follow-up and the Benz IPD analysis shifts interpretation towards increased first-inappropriate-shock risk.^8 9 13^ The contribution of this review is to integrate these developments by endpoint, horizon and design, without treating linked reports as independent evidence.

Avoidance of intravascular and intracardiac leads provides a plausible explanation for the lead-safety findings.^5 7 9^ However, complications requiring invasive intervention are not synonymous with complications confined to implantation.^19^ ATLAS’s 6-month lead-safety composite, PRAETORIAN’s parent-trial outcomes and PRAETORIAN-XL’s competing-risk estimates address different endpoints and horizons and were therefore not pooled.^8 9 19^ Individual procedural components were much sparser: only six randomised pneumothoraces and two isolated tamponades were reported. The exact pneumothorax test did not provide a relative-effect CI, and absence of S-ICD events should not be interpreted as zero risk.

The lead-related findings support the rationale for avoiding transvenous hardware. Transvenous leads are exposed to repetitive mechanical stress from cardiac motion, venous structures and traversal of the tricuspid valve, predisposing them to displacement, conductor fracture and insulation failure.^26^ S-ICD systems avoid these intracardiac stresses, although they remain vulnerable to subcutaneous lead displacement, fracture, sensing abnormalities and revision. Such complications may be less complex to manage because extraction of an intracardiac lead is generally unnecessary.^5 6^ The observed lead-related advantage should not be interpreted as proof of superior overall lead durability across every failure mechanism.

The extravascular design may introduce a different local complication profile rather than eliminating device-related morbidity. The supportive matched-cohort synthesis concerned device-related infection rather than a uniformly defined pocket-infection endpoint and remained imprecise across sparse-event models.^21–23^ Omission of Brouwer (2018) reversed the point-estimate direction, further limiting claims of increased infection risk. Non-infectious pocket bleeding was assessed separately and should not be conflated with infection.

The clinical consequences of infection may also differ between the two systems. TV-ICD infection can involve the intravascular or intracardiac lead and often necessitates transvenous lead extraction, whereas S-ICD infection is generally confined to the generator pocket or subcutaneous lead system.^4 27 28^ PRAETORIAN’s five systemic infections all occurred with TV-ICD, but this sparse evidence does not establish that S-ICD eliminates systemic infection.^19^ Thus, event frequency alone may not fully capture differences in severity, treatment complexity, procedural risk and resource use. Future studies should distinguish superficial wound complications, pocket infections, systemic infections and infections requiring complete system extraction.

Patient-centred outcomes remain insufficiently studied. ATLAS and PRAETORIAN did not identify statistically clear between-device differences in the reported device-acceptance, functional-capacity and health-related quality of life comparisons, but different instruments, assessment times and incomplete questionnaire data limit interpretation.^24 25^ These findings do not establish equivalence or show that differences in shock exposure have no patient-perceived consequences. Future comparative studies should incorporate standardised measures of pain, anxiety, device acceptance, body image, functional status and health-related quality of life.

Sensing and therapy delivery remain important considerations when selecting an S-ICD. The IPD analysis associated S-ICD with more inappropriate shocks attributed to cardiac oversensing and electromagnetic interference/myopotentials, but fewer attributed to atrial arrhythmias.^13^ These are mechanism-specific inappropriate-shock outcomes, not interchangeable measures of all sensing abnormalities. The opposing directions also show why a single aggregate sensing endpoint can obscure clinically relevant differences.

Screening, sensing algorithms and device programming remain potential modifiers of inappropriate-shock risk.^29–31^ However, improvements in these areas do not establish that the higher first-inappropriate-shock hazard observed in the randomised IPD analysis has been eliminated.^13^ The contribution of implant era, sensing configuration and programming to differences between studies could not be isolated.

The trial-level randomised estimate suggested a higher hazard of first appropriate shock with S-ICD, although the modified Hartung-Knapp sensitivity interval was markedly wider and crossed the null.^9 20^ First appropriate shock is not a measure of total shock burden or episode-level arrhythmia-termination efficacy. TV-ICDs can terminate ventricular tachycardia with ATP before shock delivery, whereas S-ICDs rely on shock therapy alone. Consequently, appropriate shock frequency reflects both arrhythmia occurrence and differences in available treatment pathways. The absence of ATP and chronic bradycardia pacing remains important for patients with recurrent monomorphic ventricular tachycardia or anticipated pacing requirements.^1 2^

Subsequent pacing and upgrade requirements are another component of this trade-off. In PRAETORIAN-XL, device changes for bradycardia pacing or ATP occurred in S-ICD recipients, while CRT-D upgrades occurred in both groups.^8^ These counts reflect device capabilities and subsequent management, not directly comparable rates of newly developed pacing or ATP indications. They inform counselling about possible future procedures, but do not establish a causal difference in the underlying need for pacing.

The mortality estimates were compatible with clinically important benefit or harm, and certainty was very low.^7 9^ Neither an I^2^ estimate of zero nor a non-significant treatment effect establishes equivalent survival. PRAETORIAN-XL extends observation but does not provide independent replication and remains vulnerable to selective participation in extended follow-up.^8^

Random-effects modelling does not make different endpoints, follow-up horizons or effect measures interchangeable. Accordingly, Cox HRs, competing-risk sHRs and source-specific risks were kept distinct. The shock design comparisons did not establish a clear first-inappropriate-shock interaction; the first-appropriate-shock interaction was method-sensitive. Patient characteristics, follow-up, implant era and programming may contribute to design differences, but these aggregate comparisons cannot identify their causes or establish patient-level effect modification. Randomised evidence therefore remained the basis for interpretation.

Device selection consequently involves balancing avoidance of intravascular leads against inappropriate-shock exposure, pacing and ATP capabilities, potential future upgrades, infection severity and patient preferences. The evidence does not establish one device strategy as superior for all patients. Established pacing requirements remain central to selection, while the low or very low certainty of several comparisons should be communicated explicitly.^1 2^ This review does not establish age-specific or aetiology-specific superiority.

### Limitations

This study has several limitations. First, only two independent randomised trials contributed to parent-trial time-to-event syntheses; modified Hartung-Knapp intervals were wide, and zero estimated heterogeneity does not establish identical underlying effects. Second, PRAETORIAN-XL involved selective non-participation in extended follow-up and is not independent of its parent trial.^8^ Third, observational estimates remain susceptible to confounding and missing data; matched-arm counts do not recover within-pair covariance, and possible cross-cohort overlap prevented a verified review-wide unique-participant total. Fourth, published HRs enabled time-to-event synthesis, but use of published IPD findings rather than individual records limited independent assessment of age-specific, aetiology-specific and follow-up-specific effects. Fifth, differences in endpoint definitions, infection severity, assessment times, implantation technique and programming limited pooling and applicability. Mixed/reimplantation populations and incompletely documented ECG screening among observational comparators further limit applicability to patients eligible for both devices. Sixth, rare events and incomplete patient-reported data restricted precision, and small evidence sets precluded informative funnel-plot/Egger testing. Analyses used nominal inference without multiplicity adjustment, so isolated p values require caution. Finally, the analytical hierarchy and sensitivity methods were refined during revision rather than prospectively prespecified, and the review did not compare S-ICD with substernal extravascular systems.

## Conclusion

Randomised evidence suggests fewer lead-related and invasive device complications with S-ICD, but a higher hazard of first inappropriate shock. First-appropriate-shock inference was method-sensitive, while mortality and rare-event comparisons remained uncertain. Certainty was low or very low, and longer-term extension findings require caution. Device selection should consider pacing and ATP requirements, future procedures, infection severity and patient preferences, without inferring equivalence from non-significant results.

## Supplementary material

10.1136/openhrt-2026-004443 online supplemental figure 1

10.1136/openhrt-2026-004443 online supplemental figure 2

10.1136/openhrt-2026-004443 online supplemental figure 3

10.1136/openhrt-2026-004443 online supplemental figure 4

10.1136/openhrt-2026-004443 online supplemental figure 5

10.1136/openhrt-2026-004443 online supplemental figure 6

10.1136/openhrt-2026-004443 online supplemental figure 7

10.1136/openhrt-2026-004443 online supplemental figure 8

10.1136/openhrt-2026-004443 online supplemental figure 9

10.1136/openhrt-2026-004443 online supplemental figure 10

10.1136/openhrt-2026-004443 online supplemental file 1

10.1136/openhrt-2026-004443 online supplemental material 1

## Data Availability

Data are available upon reasonable request. All data relevant to the study are included in the article or uploaded as supplementary information.
