# Supplementary material for: Subcutaneous versus transvenous implantable cardioverter-defibrillator therapy: an updated systematic review and meta-analysis
Source: Open Heart. 2026 Oct 20;13(2):e004443. doi: 10.1136/openhrt-2026-004443 (PMC13684192; doi:10.1136/openhrt-2026-004443)
Supplement: Supplementary file 1 [file openhrt-13-2-s007.pdf]

Risk of bias domains

Study	D1	D2	D3	D4	D5	Overall
PRAETORIAN (2020) - First inappropriate shock	+	-	-	-	+	-
ATLAS (2022) - First inappropriate shock	+	-	-	-	+	-
PRAETORIAN (2020) - First appropriate shock	+	-	-	-	+	-
ATLAS (2022) - First appropriate shock	+	-	-	-	+	-
PRAETORIAN (2020) - All-cause mortality	+	-	-	+	+	-
ATLAS (2022) - All-cause mortality	+	-	-	+	+	-
PRAETORIAN (2020/2022) - All device-related complications	+	-	-	-	+	-
PRAETORIAN-XL (2025) - All device-related complications	+	-	X	-	-	X
PRAETORIAN (2020/2022) - Lead-related complications	+	-	-	-	+	-
ATLAS (2022) - Perioperative lead-safety composite (6 months)	+	+	-	-	+	-
PRAETORIAN-XL (2025) - Lead-related complications	+	-	X	-	-	X
PRAETORIAN (2022) - Complications requiring invasive intervention	+	-	-	-	-	-
PRAETORIAN-XL (2025) - Major/invasive device complications	+	-	X	-	-	X
PRAETORIAN (2022) - Acute device-related complications	+	-	-	-	-	-
PRAETORIAN (2022) - Device-related infection	+	-	-	-	+	-
PRAETORIAN (2022) - Systemic device-related infection	+	-	-	-	-	-
ATLAS (2022) - Device infection requiring surgery (6 months)	+	+	-	-	+	-
PRAETORIAN (2022) - Early pocket bleeding component	+	-	-	-	-	-
ATLAS (2022) - Significant wound haematoma (6 months)	+	+	-	-	+	-
PRAETORIAN (2022) - Pneumothorax component	+	-	-	-	+	-
ATLAS (2022) - Pneumothorax component (6 months)	+	+	-	-	+	-
PRAETORIAN (2022) - Isolated tamponade component	+	-	-	-	+	-
PRAETORIAN-XL (2025) - All-cause mortality	+	-	X	+	-	X
PRAETORIAN (2020) - Crossover to other study device	+	-	-	-	+	-
ATLAS (2022) - ICD/lead revision, replacement or removal	+	-	-	-	+	-
PRAETORIAN QoL (2024) - DASI (30 months)	+	-	-	-	-	-
PRAETORIAN QoL (2024) - SF-36 bodily pain (30 months)	+	-	-	-	-	-
PRAETORIAN QoL (2024) - SF-36 mental health (30 months)	+	-	-	-	-	-
ATLAS QoL (2024) - FPAS acceptance (6 months)	+	-	-	-	-	-
ATLAS QoL (2024) - SF-36 physical summary (6 months)	+	+	-	-	+	-
ATLAS QoL (2024) - SF-36 mental summary (6 months)	+	+	-	-	+	-

Domains:
D1: Bias arising from the randomization process.
D2: Bias due to deviations from intended intervention.
D3: Bias due to missing outcome data.
D4: Bias in measurement of the outcome.
D5: Bias in selection of the reported result.

Judgement
X High
- Some concerns
+ Low
