# Supplementary material for: Subcutaneous versus transvenous implantable cardioverter-defibrillator therapy: an updated systematic review and meta-analysis
Source: Open Heart. 2026 Oct 20;13(2):e004443. doi: 10.1136/openhrt-2026-004443 (PMC13684192; doi:10.1136/openhrt-2026-004443)
Supplement: Supplementary file 2 [file openhrt-13-2-s009.pdf]

Risk of bias domains

Study	D1	D2	D3	D4	D5	D6	D7	Overall
Palmisano 2021 - surgical-revision complications	-	+	+	+	+	-	-	-
Palmisano 2021 - inappropriate shock	X	+	+	+	+	-	-	X
Palmisano 2021 - all-cause mortality	-	+	+	+	+	+	-	-
Palmisano 2021 - device infection	-	+	+	+	+	-	-	-
Palmisano 2021 - pocket haematoma	-	+	+	+	+	-	-	-
Palmisano 2021 - lead failure	-	+	+	+	+	-	-	-
Palmisano 2021 - lead dislodgement	-	+	+	+	+	-	-	-
Palmisano 2021 - pneumothorax	-	+	+	+	+	-	-	-
Brouwer 2018 - invasive complications (matched)	-	-	+	+	X	-	-	X
Brouwer 2018 - all-cause mortality (matched)	-	-	+	+	X	+	-	X
Brouwer 2018 - inappropriate shock (matched)	X	-	+	+	X	-	-	X
Brouwer 2018 - appropriate shock (matched)	X	-	+	+	X	-	-	X
Brouwer 2018 - device infection (matched)	-	-	+	+	X	-	-	X
Brouwer 2018 - haematoma/haemorrhage (matched)	X	-	+	+	X	-	-	X
Brouwer 2018 - lead category (matched)	-	-	+	+	X	-	-	X
Brouwer 2018 - lead category (stratified)	X	-	+	+	-	-	-	X
Brouwer 2018 - procedural pneumothorax (matched)	-	-	+	+	X	-	-	X
Brouwer 2018 - perforation with tamponade (matched)	-	-	+	+	X	-	-	X
Lee 2025 - device-related complications	X	X	+	+	?	-	-	X
Lee 2025 - inappropriate shock	X	X	+	+	?	-	-	X
Lee 2025 - appropriate shock	X	X	+	+	?	-	-	X
Lee 2025 - all-cause mortality	X	X	+	+	?	+	-	X
Lee 2025 - device infection	X	X	+	+	?	-	-	X
Lee 2025 - pocket haematoma	X	X	+	+	?	-	-	X
Brouwer 2016 - 5-year lead complications	X	+	+	+	-	-	-	X
Brouwer 2016 - appropriate shock (5-year report)	X	+	+	+	-	-	-	X
Brouwer 2016 - inappropriate shock (5-year report)	X	+	+	+	-	-	-	X
Honarbakhsh 2017 - inappropriate shock	X	+	+	+	?	-	-	X
Honarbakhsh 2017 - device infection	-	+	+	+	?	-	-	?
Honarbakhsh 2017 - acute implant-related complications	-	+	+	+	?	-	-	?
Honarbakhsh 2017 - RV lead displacement	-	+	+	+	?	-	-	?
Honarbakhsh 2017 - perforation with tamponade	-	+	+	+	?	-	-	?
Liang 2019 - procedural lead dislodgement	X	+	+	+	?	-	-	X
Liang 2019 - lead fracture during follow-up	X	+	+	+	?	-	-	X
Friedman 2016 - any in-hospital complication vs SC-TV	-	+	+	+	-	-	-	-
Friedman 2016 - any in-hospital complication vs DC-TV	-	+	+	+	-	-	-	-
Friedman 2016 - lead dislodgement vs SC-TV	-	+	+	+	-	-	-	-
Friedman 2016 - lead dislodgement vs DC-TV	-	+	+	+	-	-	-	-
Friedman 2016 - pneumothorax vs SC-TV	-	+	+	+	-	-	-	-
Friedman 2016 - pneumothorax vs DC-TV	-	+	+	+	-	-	-	-
Friedman 2016 - tamponade vs DC-TV	-	+	+	+	-	-	-	-

Domains:
D1: Bias due to confounding.
D2: Bias due to selection of participants.
D3: Bias in classification of interventions.
D4: Bias due to deviations from intended interventions.
D5: Bias due to missing data.
D6: Bias in measurement of outcomes.
D7: Bias in selection of the reported result.

Judgement
X Serious
- Moderate
+ Low
? No information
