# Supplementary material for: Subcutaneous versus transvenous implantable cardioverter-defibrillator therapy: an updated systematic review and meta-analysis
Source: Open Heart. 2026 Oct 20;13(2):e004443. doi: 10.1136/openhrt-2026-004443 (PMC13684192; doi:10.1136/openhrt-2026-004443)
Supplement: Supplementary file 4 [file openhrt-13-2-s011.docx]

# **Subcutaneous Versus Transvenous Implantable Cardioverter-Defibrillator Therapy: An Updated Systematic Review and Meta-Analysis**

**Supplementary Table S1. Summary of findings and GRADE certainty of evidence.**

**Part A. Randomised evidence: parent-trial and harmonised IPD results.**

| **Outcome** | **Relative / comparative effect (95% CI)** | **Absolute effect** | **Participants** | **Certainty assessment** | **Overall certainty** |
| --- | --- | --- | --- | --- | --- |
| **First inappropriate shock IPD; median 3.5 years** | HR 1.61 (1.06 to 2.45) | 33 more per 1,000 at 4 years (96 vs 63); point difference | 1,342 (2 RCTs; IPD) | Downgraded one level for risk of bias because of modified-ITT exclusions, incomplete rhythm adjudication and unblinded outcome assessment, and one for imprecision: 93 first events support an interval compatible with a small or a substantially larger increase. Trial-level modified-HK sensitivity was considerably less precise. | **Low** ⊕⊕◯◯ |
| **First appropriate shock Parent-trial follow-up** | REML/Wald HR 1.54 (1.14 to 2.09) | PRAETORIAN: 77 more per 1,000 at 4 years (192 vs 115); not a pooled absolute effect | 1,342 (2 RCTs) | Downgraded one level for risk of bias, including incomplete rhythm adjudication, and one for imprecision in the magnitude of the increase. The conventional interval permits modest to substantial additional shock exposure; modified-HK sensitivity gives HR 1.54 (0.22 to 10.87). | **Low** ⊕⊕◯◯ |
| **All-cause mortality Parent-trial follow-up** | REML/Wald HR 1.22 (0.89 to 1.66) | PRAETORIAN: 33 more per 1,000 at 4 years (164 vs 131); not a pooled absolute effect | 1,342 (2 RCTs) | Downgraded one level for risk of bias because of modified-ITT exclusions and incomplete follow-up, and two for very serious imprecision: the interval permits clinically important mortality benefit and harm. Modified-HK sensitivity is substantially wider. Equivalent survival is not established. | **Very low** ⊕◯◯◯ |
| **Major lead-related perioperative complications ATLAS; 6 months** | Published RD -4.4 percentage points (-6.9 to -1.9) | 44 fewer per 1,000 (69 to 19 fewer); published rounded RD | 503 (1 RCT) | Downgraded one level for risk of bias because of follow-up and unblinded component-assessment concerns, and one for imprecision: only 13 events support the large estimated reduction. Although the interval excludes a trivial absolute benefit, sparse information limits confidence in its magnitude. | **Low** ⊕⊕◯◯ |
| **Lead-related complications PRAETORIAN; 48 months** | HR 0.24 (0.10 to 0.54) | 52 fewer per 1,000 (14 vs 66); point difference | 849 (1 RCT) | Downgraded one level for risk of bias and one for imprecision: the large estimated reduction is based on 36 affected patients and a relative interval spanning 0.10 to 0.54. An important benefit is supported, but its magnitude remains vulnerable to sparse-event uncertainty. | **Low** ⊕⊕◯◯ |
| **Device complications requiring invasive intervention PRAETORIAN; 48 months** | HR 0.59 (0.35 to 0.99) | 40 fewer per 1,000 (43 vs 83); point difference | 849 (1 RCT) | Downgraded one level for risk of bias, including unblinded intervention decisions and incomplete follow-up, and one for imprecision: the interval permits a substantial reduction or almost no reduction. The outcome is defined by invasive intervention, not by procedural timing alone. | **Low** ⊕⊕◯◯ |
| **Device infection requiring surgery ATLAS; 6 months** | 2/251 vs 1/252; no pooled relative effect | 4.0 more per 1,000; crude point difference | 503 (1 RCT) | Downgraded one level for risk of bias and two for very serious imprecision because only three infections occurred. Clinically important benefit and harm remain unresolved. This six-month surgery-defined endpoint is distinct from longer-term infection requiring extraction. | **Very low** ⊕◯◯◯ |

Notes: Absolute differences are S-ICD minus TV-ICD. Published fixed-horizon risks or descriptive component-count differences are shown; the latter do not account for censoring. Except for the published ATLAS lead-safety RD, confidence intervals for the displayed direct absolute differences were unavailable. PRAETORIAN-only absolute effects accompanying two-trial HRs are illustrative source comparisons, not pooled absolute effects.

**Part A (continued). Randomised infection and rare procedural outcomes.**

| **Outcome** | **Relative / comparative effect (95% CI)** | **Absolute effect** | **Participants** | **Certainty assessment** | **Overall certainty** |
| --- | --- | --- | --- | --- | --- |
| **Device infection requiring extraction PRAETORIAN; parent follow-up** | 4/426 vs 8/423; no separate published HR retained | 9.5 fewer per 1,000; crude point difference | 849 (1 RCT) | Downgraded one level for risk of bias and two for very serious imprecision because only 12 patients were affected and no sufficiently precise time-to-event comparison was available. Crude proportions do not account for censoring or provide fixed-horizon cumulative risks. | **Very low** ⊕◯◯◯ |
| **Systemic device-related infection PRAETORIAN; 48 months** | 0 vs 5 affected patients; no finite published HR | 12 fewer per 1,000 (0 vs 12); point difference | 849 (1 RCT) | Downgraded one level for risk of bias and two for very serious imprecision. Only five systemic infections occurred, all with TV-ICD, leaving substantial uncertainty in the size of any reduction. Systemic infection is distinct from local infection and non-infectious pocket morbidity. | **Very low** ⊕◯◯◯ |
| **Pneumothorax Source-specific procedural windows** | ATLAS 0/251 vs 2/252; PRAETORIAN 0/426 vs 4/423; no pooled RR | ATLAS: 7.9 fewer/1,000; PRAETORIAN: 9.5 fewer/1,000; descriptive component-count differences | 1,352 (2 RCTs) | Downgraded one level for risk of bias and two for very serious imprecision because only six events occurred. The exact association test does not quantify the precision of the comparative effect. Trial-specific severity thresholds and procedural observation windows also differ. | **Very low** ⊕◯◯◯ |
| **Isolated cardiac tamponade Source-specific procedural windows** | ATLAS 0/251 vs 0/252; PRAETORIAN 0/426 vs 2/423; no pooled relative effect | ATLAS: 0; PRAETORIAN: 4.7 fewer/1,000; descriptive component-count differences | 1,352 (2 RCTs) | Downgraded one level for risk of bias and two for very serious imprecision because only two events occurred. ATLAS provides a double-zero comparison for isolated tamponade. The broader ATLAS perforation/pericarditis composite is not included in this outcome. | **Very low** ⊕◯◯◯ |

**Part B. Long-term randomised extension, reported separately.**

| **Outcome** | **Relative / comparative effect (95% CI)** | **Absolute effect** | **Participants** | **Certainty assessment** | **Overall certainty** |
| --- | --- | --- | --- | --- | --- |
| **All-cause mortality PRAETORIAN-XL; 8 years** | Published sHR 1.03 (0.81 to 1.32) | 4 more per 1,000 (263 vs 259); point difference | 849 parent mITT; 528 extension consenters | Downgraded two levels for risk of bias because extended follow-up was affected by substantial, prognostically selective nonparticipation, and one for imprecision: the interval permits relevant benefit or harm. The near-null point estimate does not establish equivalent survival. | **Very low** ⊕◯◯◯ |
| **Lead-related complications PRAETORIAN-XL; 8 years** | sHR 0.33 (0.17 to 0.63) | 59 fewer per 1,000 (24 vs 83); point difference | 849 parent mITT; 528 extension consenters | Downgraded two levels for risk of bias because of prognostically selective extension nonparticipation, and one for imprecision in the magnitude of a large reduction supported by sparse lead-related events. The summary sHR is not a constant effect throughout follow-up. | **Very low** ⊕◯◯◯ |
| **Major/invasive device complications PRAETORIAN-XL; 8 years** | sHR 0.58 (0.36 to 0.95) | 45 fewer per 1,000 (57 vs 102); point difference | 849 parent mITT; 528 extension consenters | Downgraded two levels for risk of bias because of selective extension nonparticipation, and one for imprecision: the interval permits a large reduction or little reduction. Non-proportional hazards also qualify interpretation of the overall long-term sHR. | **Very low** ⊕◯◯◯ |

PRAETORIAN-XL extends the same parent cohort and is not an additional RCT. The 528 extension consenters are not added to the parent sample or used as denominators for all accumulated events. Published sHRs are retained separately from Cox HRs; no ordinary proportional-hazards absolute conversion is applied. For PRAETORIAN early components, the acute classification refers to the last implanted device.

**Part C. Observational evidence, assessed separately from randomised evidence.**

| **Outcome** | **Relative / comparative effect (95% CI)** | **Absolute effect** | **Participants** | **Certainty assessment** | **Overall certainty** |
| --- | --- | --- | --- | --- | --- |
| **First inappropriate shock Core matched cohorts** | Brouwer: HR 1.49 (0.94 to 2.37); Lee: HR 2.449 (0.151 to 39.723); not pooled | No aligned fixed-horizon absolute effect | 821 (2 cohorts) | Downgraded two levels for risk of bias because of residual confounding, participant selection and missing data, and one for imprecision. The intervals permit little effect or a substantial increase. Neither core observational HR remains after restriction to Low/Moderate ROBINS-I risk. | **Very low** ⊕◯◯◯ |
| **First appropriate shock Core matched cohorts** | Brouwer: HR 0.62 (0.40 to 0.96); Lee: HR 1.562 (0.446 to 5.472); not pooled | No aligned fixed-horizon absolute effect | 821 (2 cohorts) | Downgraded two levels for risk of bias and one for imprecision. Point estimates differ in direction, but the wide, overlapping intervals do not establish inconsistency independently of sampling uncertainty. The exploratory RCT/NRS interaction cannot identify the cause of the discrepancy. | **Very low** ⊕◯◯◯ |
| **All-cause mortality Core matched cohorts** | Brouwer: HR 0.74 (0.41 to 1.35); Palmisano: 3/169 vs 3/169; Lee: 0/13 vs 2/26; not pooled | Source-specific crude counts only; no pooled absolute effect | 1,159 (3 cohorts) | Downgraded two levels for risk of bias and one for imprecision. The published HR permits benefit and harm; the other cohorts contribute few deaths at nonidentical follow-up. These crude counts are not equivalent to a common time-to-event estimate. | **Very low** ⊕◯◯◯ |
| **Mechanical lead-related complications Brouwer; 3 years** | Adjusted HR 0.18 (0.07 to 0.50) | No aligned absolute effect for the adjusted population | 2,387 analysed (1 stratified cohort comparison) | Downgraded two levels for risk of bias and one for imprecision in the magnitude of a large, sparse-event association: the HR interval spans 0.07 to 0.50. Residual confounding precludes upgrading for effect size. The stratified population overlaps the matched subset. | **Very low** ⊕◯◯◯ |
| **Complications requiring invasive/surgical revision Source-specific comparisons** | Palmisano: OR 0.16 (0.05 to 0.56); Brouwer: HR 1.33 (0.79 to 2.24); not pooled | Palmisano: 3/169 vs 17/169; no shared fixed-horizon absolute effect | 1,120 (2 cohorts) | Downgraded two levels for risk of bias, one for indirectness because severity and ascertainment frameworks differ, and one for imprecision. The source-specific OR and HR address related, nonidentical constructs and were not pooled. | **Very low** ⊕◯◯◯ |
| **Device-related infection Core matched cohorts** | MH RR 2.06 (0.78 to 5.42); supportive marginal-arm sensitivity | No pooled fixed-horizon absolute effect; source-specific differences retained | 1,159 (3 cohorts) | Downgraded two levels for risk of bias, one for inconsistency because estimates oppose one another and exclusion of Brouwer reverses the pooled direction, and two for very serious imprecision. All alternative methods remain highly uncertain; arm totals do not recover matched-pair adjustment. | **Very low** ⊕◯◯◯ |
| **Pneumothorax Core matched cohorts** | Palmisano 0/169* vs 1/169; Brouwer 0/391 vs 1/391; no pooled RR | 5.9 and 2.6 fewer per 1,000, respectively; crude point differences | 1,120 (2 cohorts) | Downgraded two levels for risk of bias and two for very serious imprecision because only two events were reported. Palmisano's S-ICD dash was interpreted as zero. Neither that assumption nor the exact association test establishes equivalence. | **Very low** ⊕◯◯◯ |
| **Perforation with tamponade Core matched source** | Brouwer 0/391 vs 1/391; no pooled relative effect | 2.6 fewer per 1,000; crude point difference | 782 (1 cohort) | Downgraded two levels for risk of bias and two for very serious imprecision because only one event was reported. The source-defined perforation-with-tamponade component remains separate from other pericardial endpoints; no pooled relative effect was estimated. | **Very low** ⊕◯◯◯ |

GRADE: Randomised evidence and observational evidence assessed with ROBINS-I begin at high certainty and are rated down for the documented limitations; non-randomisation is not penalised twice. Large observational associations were not upgraded where residual confounding remained plausible. The five domains were considered separately, without double-counting the same concern. Certainty: high (⊕⊕⊕⊕), moderate (⊕⊕⊕◯), low (⊕⊕◯◯), very low (⊕◯◯◯).

Imprecision: Review-specific absolute benchmarks were 20 per 1,000 for first shocks; 10 per 1,000 for mortality, lead-related and major/invasive complications; 5 per 1,000 for infection and pneumothorax; and 2 per 1,000 for tamponade, at the stated horizons. These post-review judgement benchmarks are not validated minimum important differences or noninferiority margins. Confidence-interval position and available event information were considered together; sparse information could warrant downgrading even where a large effect excluded a trivial benefit.

Publication bias was assessed qualitatively from the available reports and protocols. No additional downgrade was applied, but publication bias cannot be excluded. Funnel-plot asymmetry/Egger tests were not performed for these small evidence sets. *Palmisano's S-ICD pneumothorax cell was a dash, interpreted as zero. Overlapping populations and repeated outcomes are not summed.

**Supplementary Table S2. Sensitivity analyses for primary and additional outcomes.**

**Part A. Dataset and analytic sensitivity analyses: time-to-event outcomes.**

| **Outcome** | **Analysis** | **Studies (k)** | **Effect estimate (95% CI)** | **I^2^** | **P value** | **Interpretation** |
| --- | --- | --- | --- | --- | --- | --- |
| **First inappropriate shock Randomised** | Published harmonised IPD synthesis (reference) | 2 | HR 1.61 (1.06 to 2.45) | NA | 0.030 | Primary randomised synthesis. The two parent trials are not entered again as independent IPD studies. |
| **First inappropriate shock Randomised** | Trial-level REML with Wald inference | 2 | HR 1.60 (1.05 to 2.43) | 0% | 0.028 | Published Cox HRs were synthesised; no crude RR or overlapping extension was added. |
| **First inappropriate shock Randomised** | Same REML model; modified Hartung-Knapp sensitivity | 2 | HR 1.60 (0.11 to 24.13) | 0% | 0.271 | The point estimate was unchanged, but the t-based interval was markedly wider and included 1. |
| **First appropriate shock Randomised** | Trial-level REML with Wald inference | 2 | HR 1.54 (1.14 to 2.09) | 0% | 0.005 | Published Cox HRs were synthesised; no crude RR or overlapping extension was added. |
| **First appropriate shock Randomised** | Same REML model; modified Hartung-Knapp sensitivity | 2 | HR 1.54 (0.22 to 10.87) | 0% | 0.217 | The point estimate was unchanged, but the t-based interval was markedly wider and included 1. |
| **All-cause mortality Randomised** | Trial-level REML with Wald inference | 2 | HR 1.22 (0.89 to 1.66) | 0% | 0.222 | Published Cox HRs were synthesised; no crude RR or overlapping extension was added. |
| **All-cause mortality Randomised** | Same REML model; modified Hartung-Knapp sensitivity | 2 | HR 1.22 (0.16 to 9.24) | 0% | 0.437 | Both intervals included 1; modified-HK inference showed substantially greater uncertainty. |
| **All three parent-trial HR outcomes** | Common-effect/Wald sensitivity | 2 each | Identical to the corresponding REML/Wald result | 0% | Same | REML estimated τ^2^=0 for each outcome, giving identical common-effect and REML/Wald results. |

Notes: Treatment effects are oriented S-ICD/TV-ICD. The published Benz IPD result is not pooled again with its two parent trials. Trial-level time-to-event results use REML/Wald, with modified-Hartung-Knapp and common-effect sensitivities. These analytical choices were specified during revision, not before the original review analyses.

The same estimated between-study variance and point estimate are retained for each Wald/modified-HK comparison. Modified HK uses q* = max(1, q) and a t reference distribution: df=1 for two-trial treatment estimates. A zero heterogeneity estimate does not establish identical underlying effects.

**Supplementary Table S2 (continued). Part A. Sparse-event models.**

| **Outcome** | **Analysis** | **Studies (k)** | **Effect estimate (95% CI)** | **I^2^** | **P value** | **Interpretation** |
| --- | --- | --- | --- | --- | --- | --- |
| **Device-related infection Matched cohorts** | Mantel-Haenszel RR; no continuity correction | 3 | RR 2.06 (0.78 to 5.42) | NA | 0.145 | Marginal matched-arm analysis; the interval included 1. Pair-level covariance was unavailable and residual confounding was not removed. |
| **Device-related infection Matched cohorts** | REML/Wald sensitivity | 3 | RR 1.465 (0.305 to 7.026) | 45% | 0.633 | The point estimate was attenuated and uncertainty increased. This remains an unadjusted count-based sensitivity. |
| **Device-related infection Matched cohorts** | REML with modified Hartung-Knapp | 3 | RR 1.465 (0.047 to 45.765) | 45% | 0.680 | The interval was extremely wide; the same REML estimate and weights were retained. |
| **Device-related infection Matched cohorts** | Binomial random-slope GLMM; Wald inference | 3 | OR 2.05 (0.78 to 5.37) | NA | 0.143 | The random-slope variance was estimated at zero; the point estimate equals the stratified common-effect logistic estimate. |
| **Device-related infection Matched cohorts** | Same GLMM; profile-likelihood interval / LR test | 3 | OR 2.05 (0.32 to 5.68) | NA | 0.235 | Approximate χ² profile-likelihood inference; study intercepts and the variance component were re-estimated at each fixed treatment effect. |
| **Pocket haematoma/bleeding Randomised** | Mantel-Haenszel RR; component-count sensitivity | 2 | RR 3.32 (0.92 to 12.02) | NA | 0.067 | Exploratory component-count analysis. Severity criteria and observation windows differ between trials; the component-count assumptions are stated below. |
| **Pocket haematoma/bleeding Randomised** | REML/Wald sensitivity | 2 | RR 3.32 (0.92 to 12.01) | 0% | 0.068 | The pooled point estimate and Wald interval were essentially unchanged; source definition and timing limitations remain. |
| **Pocket haematoma/bleeding Randomised** | REML with modified Hartung-Knapp | 2 | RR 3.32 (0.00079 to 13,883) | 0% | 0.319 | The t-based interval was extremely wide despite an unchanged point estimate; only two trials contribute. |
| **Pocket haematoma/bleeding Matched cohorts** | Mantel-Haenszel RR; no continuity correction | 3 | RR 0.45 (0.11 to 1.78) | NA | 0.255 | Lee's single-zero S-ICD arm was retained without correction. All three cohort point estimates were below 1. |

The logistic GLMM estimates an OR; the MH and inverse-variance count analyses estimate RRs. Matched-arm totals do not recover within-pair covariance or remove residual confounding. The GLMM uses fixed study intercepts and an S-ICD-coded random slope. The point estimate is at τ^2^=0, but nuisance variance is re-estimated for the profile interval; Wald and χ^2^ profile/LR inference are approximate.

Pocket bleeding is an exploratory component-count sensitivity: ATLAS observes six months, whereas PRAETORIAN classifies early events within 30 days of the last implanted device. Binomial analyses of PRAETORIAN components assume that each tabulated event represents a different affected patient; patient-level recurrence cannot be checked from aggregate tables. Infection is excluded from the bleeding endpoint.

**Supplementary Table S2 (continued). Part A. Zero-event handling and design comparisons.**

| **Outcome** | **Analysis** | **Studies (k)** | **Effect estimate (95% CI)** | **I^2^** | **P value** | **Interpretation** |
| --- | --- | --- | --- | --- | --- | --- |
| **Pneumothorax Randomised** | Exact conditional stratified test; no corrected RR | 2 | No pooled relative-effect CI | NA | 0.031 | ATLAS: 0/251 vs 2/252; PRAETORIAN: 0/426 vs 4/423. The exact P value does not provide an effect-size interval. |
| **Pneumothorax Matched cohorts** | Exact conditional stratified test; no corrected RR | 2 | No pooled relative-effect CI | NA | 0.500 | Only two reported events; this is a study-stratified association test, not a randomisation test. Palmisano's S-ICD dash was interpreted as zero. |
| **Isolated cardiac tamponade Randomised** | Double-zero/source-specific reporting | 2 | ATLAS 0 vs 0; PRAETORIAN 0 vs 2 | NA | NA | ATLAS remained visible as a double-zero comparison; its broader pericardial composite was not substituted for isolated tamponade. |
| **First inappropriate shock RCT vs matched cohorts** | Design moderator: modified HK (primary exploratory inference) | 2+2 | Ratio of HRs 1.06 (0.27 to 4.13) | NA | 0.871 | RCT/NRS orientation; shared residual τ^2^=0 and residual df=2. No statistically clear design interaction. |
| **First inappropriate shock RCT vs matched cohorts** | Same design moderator: Wald sensitivity | 2+2 | Ratio of HRs 1.06 (0.57 to 1.97) | NA | 0.854 | The same coefficient and variance estimate were used. Neither inferential procedure identified a statistically clear design interaction. |
| **First appropriate shock RCT vs matched cohorts** | Design moderator: modified HK (primary exploratory inference) | 2+2 | Ratio of HRs 2.25 (0.73 to 6.92) | NA | 0.090 | RCT/NRS orientation; shared residual τ^2^=0 and residual df=2. The interval included 1 and was substantially wider than the Wald interval. |
| **First appropriate shock RCT vs matched cohorts** | Same design moderator: Wald sensitivity | 2+2 | Ratio of HRs 2.25 (1.35 to 3.75) | NA | 0.002 | The Wald interval excluded 1, unlike modified HK. Evidence for a design interaction was sensitive to the inference method. |
| **Pocket haematoma/bleeding RCT vs matched cohorts** | Independent-stratum MH contrast; Wald | 2+3 | Ratio of RRs 7.38 (1.12 to 48.44) | NA | 0.037 | Exploratory contrast; sparse events, component-count assumptions and nonidentical windows limit interpretation. |

Exact P values use central two-sided conditional tests (twice the smaller tail), not relative-effect confidence intervals. No blanket continuity correction was applied. Double-zero studies remain in the evidence inventory. Conventional log-Wald failure with zero cells does not mean that all exact or profile-based intervals are impossible. Palmisano's S-ICD pneumothorax dash was interpreted as zero.

Design contrasts are ratios of treatment effects, not overall treatment effects. Each shock moderator uses four studies and two coefficients (residual df=2) and assumes one shared residual τ^2^. The primary exploratory inference uses modified HK, with Wald sensitivity. All interaction tests are nominal and unadjusted for multiplicity.

**Supplementary Table S2 (continued). Part A. Risk-of-bias restrictions.**

| **Outcome** | **Analysis** | **Studies (k)** | **Effect estimate (95% CI)** | **I^2^** | **P value** | **Interpretation** |
| --- | --- | --- | --- | --- | --- | --- |
| **Parent-trial shock / mortality** | Exclude High RoB 2 results | 2 each | Unchanged study set | 0% | Same | All six parent shock/mortality results were rated Some concerns, not High; the analysed study sets were unchanged. |
| **Device-related infection Matched cohorts** | Retain Low/Moderate ROBINS-I results | 1 | Palmisano RR 0.50 (0.046 to 5.46) | NA | NA | Palmisano alone remained. This is a single-study result, not a lower-risk meta-analysis. |
| **Pocket haematoma/bleeding Matched cohorts** | Retain Low/Moderate ROBINS-I results | 1 | Palmisano RR 0.33 (0.035 to 3.17) | NA | NA | Palmisano alone remained. This is a single-study result, not a lower-risk meta-analysis. |
| **Core observational shock HRs** | Retain Low/Moderate ROBINS-I results | 0 | Not estimable | NA | NA | Both core observational HR studies were rated Serious risk, leaving no eligible lower-risk stratum. |
| **Long-term PRAETORIAN-XL outcomes** | Exclude High RoB 2 extension results | 0 | No independent extension remains | NA | NA | The parent pools were unchanged because the extension was already presented separately; no pooled leave-extension-out estimate applies. |

Restrictions use the result-specific RoB 2 and ROBINS-I assessments. Low/Moderate-risk observational restrictions exclude Serious, Critical and No-information ratings. A single retained study is not a meta-analysis; an empty restricted set is not evidence of no effect. The long-term extension was never included in the parent-trial pools.

**Supplementary Table S2 (continued). Part B. Leave-one-out influence analysis.**

| **Outcome** | **Studies (k)** | **Full estimate (95% CI)** | **Full I^2^** | **Leave-one-out effect range** | **Leave-one-out I^2^ range** | **Conclusion stable?** | **Possible changed conclusion** | **Most influential omission** |
| --- | --- | --- | --- | --- | --- | --- | --- | --- |
| **First inappropriate shock Randomised** | 2 | HR 1.60 (1.05 to 2.43) | 0% | HR 1.43 to 2.37 | Not applicable; k=1 | No | Both omissions leave a single-trial interval including 1; point estimates remain above 1. | PRAETORIAN (precision) |
| **First appropriate shock Randomised** | 2 | HR 1.54 (1.14 to 2.09) | 0% | HR 1.52 to 1.64 | Not applicable; k=1 | No | Omitting PRAETORIAN leaves ATLAS with an interval including 1; point estimates remain above 1. | PRAETORIAN |
| **All-cause mortality Randomised** | 2 | HR 1.22 (0.89 to 1.66) | 0% | HR 1.02 to 1.23 | Not applicable; k=1 | Yes | Both single-trial CIs include 1; uncertainty remains substantial. | PRAETORIAN (precision) |
| **Device-related infection Matched cohorts** | 3 | MH RR 2.06 (0.78 to 5.42) | NA | RR 0.58 to 2.83 | NA | No | Every interval includes 1, but omission of Brouwer reverses the point direction (RR 0.58). | Brouwer 2018 |
| **Pocket haematoma/bleeding Matched cohorts** | 3 | MH RR 0.45 (0.11 to 1.78) | NA | RR 0.27 to 0.55 | NA | Yes | Every point estimate is below 1 and every interval includes 1; precision remains poor. | Brouwer 2018 (point-estimate change) |

Notes: "Conclusion stable?" requires both unchanged point-estimate direction and unchanged conventional statistical interpretation after every omission. For two-trial outcomes, omission leaves a single published trial HR, not a new meta-analysis; I^2^ is therefore not calculated. The inappropriate-shock influence display concerns the trial-level verification analysis, not a leave-one-out reanalysis of Benz IPD. I^2^ is not supplied for the correction-free MH summaries. All inference is nominal; non-significance is not equivalence.

Abbreviations: CI, confidence interval; GRADE, Grading of Recommendations Assessment, Development and Evaluation; HR, hazard ratio; ICD, implantable cardioverter-defibrillator; IPD, individual-participant data; k, number of independent studies; KM, Kaplan-Meier; LR, likelihood ratio; MH, Mantel-Haenszel; mITT, modified intention-to-treat; NA, not applicable; NRS, non-randomised study; OR, odds ratio; RD, risk difference; REML, restricted maximum likelihood; RR, risk ratio; sHR, subdistribution hazard ratio; S-ICD, subcutaneous ICD; TV-ICD, transvenous ICD.

**Supplementary Table S3. Cohort-publication linkage, analysis populations and handling of overlapping reports.**

# **A. Randomised trial families and linked publications**

## **PRAETORIAN (NCT01296022)**

## 39 centres in Europe and the United States; recruitment March 2011 to January 2017. The original randomisation comprised 876 participants (442 S-ICD; 434 TV-ICD), of whom 849 (426; 423) were included in the primary modified intention-to-treat analysis. [1]

| **Publication / report** | **Analysis population (S-ICD / TV-ICD)** | **Follow-up / assessment** | **Contribution and handling of repeated participants** |
| --- | --- | --- | --- |
| Knops et al. 2020 [1] Parent trial | 426 / 423 in the primary mITT analysis; 442 / 434 initially randomised. | Median 49.1 months; 48-month risk estimates. | Parent-trial first-shock and mortality HR inputs. The 27 post-randomisation exclusions distinguish the 849-person analysis population from the 876 initially randomised. |
| Knops et al. 2022 [2] Device-related complications | Parent mITT: 426 / 423. Event and affected-patient counts depend on the endpoint. | Parent follow-up; 48-month estimates. | Detailed complication definitions, lead-related and invasive-intervention outcomes, and procedural components. Shares the parent population; an endpoint is not entered twice through its parent and secondary reports. |
| Knops et al. 2022 [3] Appropriate therapy / ATP | Parent mITT: 426 / 423. Shock-recipient and arrhythmia-episode subsets are endpoint-specific. | Median 52 months in this report. | Source-specific clinical shock efficacy and ATP/therapy findings. Patients, episodes, shocks and ATP attempts are distinct units and do not add participants to the parent trial. |
| Olde Nordkamp et al. 2024 [4] Inappropriate therapy | Parent mITT: 426 / 423; therapy and mechanism subsets. | Median 49.1 months. | Secondary inappropriate-therapy and mechanism findings. Shares the parent and IPD populations; episode categories may overlap and are not independent patient groups. |
| Knops et al. 2024 [5] Quality of life | Parent mITT: 426 / 423. At 30 months, 293 / 289 provided at least one SF-36 subscale. | Baseline, discharge, 12 and 30 months. | Instrument- and time-specific patient-reported results, without pooling across scales. The 582 respondents are a subset, not additional participants or a universal questionnaire denominator. |
| Olde Nordkamp et al. 2025 [6] PRAETORIAN-XL | Parent mITT: 426 / 423. Extension consenters: 263 / 265. | Median approximately 87.5 months; 8-year estimates. | Long-term extension of the same trial, presented separately from parent-trial meta-analyses. The 528 consenters add no new participants and are not denominators for all accumulated events. |

Population sizes are shown in S-ICD / TV-ICD order. In randomised reports, the group labels refer to allocated strategy unless a different source-defined analysis is specified. Source references are listed in Part E.

## **ATLAS (NCT02881255)**

14 Canadian centres; recruitment February 2017 to July 2021. Of 544 enrolled participants, 503 were randomised (251 S-ICD; 252 TV-ICD); the implanted/harmonised clinical analysis population comprised 493 (247; 246). The failed-screen registry is not a further randomised cohort. [7]

| **Publication / report** | **Analysis population (S-ICD / TV-ICD)** | **Follow-up / assessment** | **Contribution and handling of repeated participants** |
| --- | --- | --- | --- |
| Healey et al. 2022 [7] Parent trial | 251 / 252 for six-month safety; 247 / 246 for the implanted/harmonised clinical population. | Six-month safety; mean 2.5 years of clinical follow-up. | Safety and clinical time-to-event results retain their source-specific denominators. The two population sizes are not interchangeable and are not added. Eligibility permitted enrolment after prior device removal. |
| Carroll et al. 2024 [8] Device acceptance / quality of life | FPAS complete cases: 207 / 197. Paired SF-36: 191 / 188. | FPAS at 1 and 6 months; SF-36 preimplant and at 6 months. | Source-specific patient-reported results. Respondents belong to ATLAS and add no participants. The FPAS adjustment uses a one-month postimplant score, not a prerandomisation baseline. |
| Leong et al. 2024 [9] TR / RV echocardiography | Parent: 251 / 252. Baseline echocardiograms: 248 / 249; six-month echocardiograms: 224 / 222. | Baseline to 6 months. | Linked post hoc randomised outcome report examined for TR/RV source attribution; not an additional trial or pooled input. Image counts do not establish the paired/evaluable denominator for every analysis. |

## **Cross-trial individual-participant-data report**

| **Publication** | **Linked trial populations** | **Follow-up** | **Contribution and handling of repeated participants** |
| --- | --- | --- | --- |
| Benz et al. 2026 [10] Published online in 2025 | PRAETORIAN 849 + ATLAS 493 = 1,342; allocated groups 673 / 669. | Median 3.5 years. | Published harmonised first-inappropriate-shock synthesis and mechanism/subgroup findings. One report links both parent trials. It is not a third trial; its estimate is not pooled again with the same parent estimates. |

The six PRAETORIAN-specific reports, three ATLAS-specific reports and one cross-trial IPD report describe two parent randomisations. Repeated publications, extension consenters, echocardiographic samples and questionnaire respondents are not summed as additional participants.

# **B. Observational comparison reports**

The study populations below are identified by their centres, registry and recruitment period. A separate published comparison does not establish that all its participants are distinct from participants in every other report.

| **Publication / comparison** | **Centres, registry and recruitment** | **Analysis population (S-ICD / TV-ICD)** | **Follow-up** | **Contribution and overlap handling** |
| --- | --- | --- | --- | --- |
| Lee et al. 2025 [11] | National Taiwan University Hospital, Taipei; January 2020 to December 2024. Registry identifier not reported. | 13 / 26 after 1:2 propensity matching; 13 / 97 before matching. | Median 2.49 / 2.84 years. | Contributes to matched-cohort infection/bleeding analyses and shock design comparisons. The unmatched cohort is not added to the matched sample. No cross-report overlap is demonstrated. |
| Palmisano et al. 2021 [12] POINTED | Seven Italian centres; NCT03612635; May 2014 to December 2019. | 169 / 169 propensity-matched; source cohort 420 / 679. | Median 30.3 / 31.3 months. | Contributes to matched infection/bleeding analyses, pneumothorax testing and source-specific outcomes. Matched/unmatched sets are nested. POINTED is distinct from the Rhythm Detect registry used by Viani. |
| Brouwer et al. 2018 [13] SIMPLE / EFFORTLESS | International SIMPLE (NCT00800384) and EFFORTLESS (NCT01085435). Exact recruitment dates not restated in the comparative report. | 391 / 391 matched; propensity-stratified analysis: 2,387 in total. | Reported 2.9 / 3.3 years; 3-year estimates. | Matched sample supplies shock, infection and procedural results; the mechanical-lead adjusted estimate uses the larger stratified population. Both analyses share the source cohort. Possible local-cohort overlap is unquantified. |
| Honarbakhsh et al. 2017 [14] | Single tertiary London centre; 2010 to 2015. Registry identifier not reported. | 69 / 69 propensity-matched from 69 / 429. | Mean 31 / 32 months. | Source-specific perforation-with-tamponade result; not pooled with Brouwer. Possible EFFORTLESS overlap is unquantified. Adult-only and wholly de novo status are not fully documented. |
| Liang et al. 2019 [15] | Mayo Clinic, United States; January 2012 to December 2016. Registry identifier not reported. | 86 / 86 directly matched. | Mean 23 / 24 months. | Source-specific observational comparison, not included in the pooled analyses. Full age range, wholly de novo status and patient-level intersections with registries are not established. |
| Brouwer et al. 2016 [16] | AMC Amsterdam (S-ICD), 2009 to 2015; LUMC Leiden (TV-ICD), 2005 to 2014. | 140 / 140 propensity-matched. | Median 3 / 5 years; 5-year KM estimates. | Source-specific long-term comparison, not pooled with SIMPLE/EFFORTLESS. PRAETORIAN participants were explicitly excluded. EFFORTLESS overlap remains possible. Six S-ICD recipients had concomitant transvenous pacemakers. |

Direct matching is distinguished from propensity-score matching. Source populations and matched, stratified or responder subsets are recorded separately; neither matching nor a separate publication establishes cross-report participant independence.

# **B. Observational comparison reports (continued)**

These additional reports are retained in the source-linkage map for population, denominator and overlap attribution. None contributes to the pooled analyses; their presence in this map does not imply inclusion in a pooled effect estimate.

| **Publication / comparison** | **Centres, registry and recruitment** | **Analysis population (S-ICD / TV-ICD)** | **Follow-up** | **Contribution and overlap handling** |
| --- | --- | --- | --- | --- |
| Viani et al. 2019 [17] Rhythm Detect | 12 Italian centres; NCT02275637; 2011 to 2017. | 90 / 139; post-extraction reimplantation cohort. | Median 17 months overall. | Wholly post-extraction population, not a de novo comparison. Distinct registry and implant setting from POINTED; shared Italian centres alone do not establish a numerical overlap. |
| Boveda et al. 2018 [18] | Clinique Pasteur, Toulouse; January to December 2016. Registry identifier not reported. | 31 / 31 age- and sex-matched. | Mean 6 months. | Mixed initial implantation/reimplantation; eight S-ICD recipients had previous ICDs. Age- and sex-matched comparison, not propensity-score matching. Not included in the pooled analyses. |
| Mithani et al. 2018 [19] | Cooper University Hospital, Camden; local NCDR ICD Registry; 22 October 2012 to 22 September 2015. | 91 / 91 directly matched. | 180 days. | Possible overlap with the national NCDR population in Friedman 2016. The matched-sample intersection is not reported; the two reports are not jointly pooled. Prior device infection/reimplantation is represented. |
| Al-Ghamdi et al. 2017 [20] | King Faisal Specialist Hospital and Research Centre, Riyadh; December 2014 to May 2016. Registry identifier not reported. | 30 / 30 comparative sample. | Mean 710.6 / 632 days. | S-ICD age range 16-64 years and previous devices are reported; not an established adult-only, de novo population. Not included in the pooled analyses. No numerical cross-report overlap is demonstrated. |
| Köbe et al. 2017 [21] | University Hospital Münster; recruitment dates not reported. Registry identifier not reported. | 42 / 42 responders; 60 / 60 invited. | Mean time since implant 622.1 / 941.9 days. | Possible overlap with the Münster component of Köbe 2013. The 30 / 30 values are male counts, not analysis denominators. Not included in the first-shock meta-analyses or design comparisons. |
| Friedman et al. 2016 [22] | US national NCDR ICD Registry; 28 September 2012 to 31 March 2015. | 1,920 S-ICD; 1,920 single-chamber and 1,920 dual-chamber TV-ICD. | Index admission only. | One 5,760-person three-arm matched population. Both TV-ICD contrasts reuse the same S-ICD arm. Possible Cooper overlap; not jointly pooled with Mithani or with longitudinal endpoints. |
| Köbe et al. 2013 [23] | Münster, Düsseldorf and Munich; exact recruitment dates not reported. Registry identifier not reported. | 69 / 69 case-matched; 53 of 69 S-ICD implants were de novo. | Mean 217 days in abstract; first visit at 4 weeks. | Not a device-strategy randomised trial. Potential Münster and EFFORTLESS intersections are unquantified. Four weeks is the first follow-up visit, not total observation. Not included in the pooled analyses. |

"Not reported" refers to the identified source report. Potential overlap is distinguished from confirmed participant reuse; no overlap count is inferred solely from shared authors, centres or recruitment years.

# **C. Analysis-specific participant accounting**

| **Outcome / analysis** | **Contributing source populations** | **Analysis denominator** | **Counting rule** |
| --- | --- | --- | --- |
| First inappropriate shock; first appropriate shock; all-cause mortality | PRAETORIAN + ATLAS | S-ICD: 673; TV-ICD: 669. Total: 1,342 (849 + 493). | Two parent clinical-analysis populations. Benz is a linked synthesis of these same participants; PRAETORIAN-XL is not an additional population. |
| Randomised procedural component comparisons and bleeding sensitivity | PRAETORIAN + ATLAS | S-ICD: 677; TV-ICD: 675. Total: 1,352 (849 + 503). | Source-specific safety denominators. This total differs from 1,342 because ATLAS uses 503 rather than 493 participants. Trial observation windows and component definitions remain distinct. |
| Matched infection and pocket bleeding syntheses | Palmisano 2021 + Brouwer 2018 + Lee 2025 | S-ICD: 573; TV-ICD: 586. Total: 1,159 (338 + 782 + 39). | The selected matched samples are used. Larger unmatched or alternative stratified samples are not added. |
| Matched shock estimates and observational part of design comparisons | Brouwer 2018 + Lee 2025 | S-ICD: 404; TV-ICD: 417. Total: 821 (782 + 39). | The same two matched sources enter the design comparison; a moderator estimate does not create another participant population. |
| Matched pneumothorax association test | Palmisano 2021 + Brouwer 2018 | S-ICD: 560; TV-ICD: 560. Total: 1,120 (338 + 782). | A stratified association test, not a pooled risk-ratio estimate. These cohorts also contribute to other outcomes and are counted only within the relevant analysis. |
| Adjusted mechanical lead-related comparison | Brouwer 2018, propensity-stratified analysis | 2,387 in total. | Alternative analysis of the SIMPLE/EFFORTLESS source population. Not added to its 782-person matched analysis. |
| Quality of life, TR/RV, pacing/upgrades and long-term follow-up | Linked trial reports | Instrument-, endpoint- and horizon-specific; see Part A. | Source-specific presentation. Questionnaire respondents, evaluable images, device changes and extension consenters do not create new study populations. |

# **D. Interpretation of the linkage map**

1. Five study comparisons contribute to at least one retained meta-analysis, including pooled sensitivity analyses: PRAETORIAN, ATLAS, Palmisano 2021, Brouwer 2018 and Lee 2025. This is a union across outcomes, not one mixed-design treatment pool.

2. The map covers 23 publications: six PRAETORIAN-specific reports, three ATLAS-specific reports, one cross-trial IPD report and 13 observational comparison reports. It describes source relationships and analytic contribution; the number mapped is not, by itself, a count of eligible studies or independent participants.

3. Known reuse is handled by linking secondary/extension reports to their parent populations and avoiding duplicate contributions within the same synthesis. Possible observational intersections that were not quantified in the source reports remain explicitly identified. No review-wide unique-participant total is obtained by adding publication sample sizes.

Abbreviations: AMC, Academic Medical Centre; ATP, antitachycardia pacing; FPAS, Florida Patient Acceptance Survey; HR, hazard ratio; ICD, implantable cardioverter-defibrillator; IPD, individual-participant data; KM, Kaplan-Meier; LUMC, Leiden University Medical Centre; mITT, modified intention-to-treat; NCDR, National Cardiovascular Data Registry; RV, right ventricle; SF-36, 36-Item Short Form Health Survey; S-ICD, subcutaneous ICD; TR, tricuspid regurgitation; TV-ICD, transvenous ICD.

# **E. Source references and verification locations**

Reference numbers below are local to this supplementary table. DOI identifiers distinguish reports with the same first author and publication year; appendices and duplicate file copies are not counted as additional reports.

| **Ref.** | **Publication** | **DOI** | **Source location** |
| --- | --- | --- | --- |
| 1 | Knops et al. 2020 | 10.1056/NEJMoa1915932 | Figure 1; Methods and Results; Tables 1-3. |
| 2 | Knops et al. 2022 (complications) | 10.1093/eurheartj/ehac496 | Methods; Tables 2-5; source-specific procedural timing. |
| 3 | Knops et al. 2022 (appropriate therapy) | 10.1161/CIRCULATIONAHA.121.057816 | Methods; Figures 1-3; participant/episode units. |
| 4 | Olde Nordkamp et al. 2024 | 10.1161/CIRCEP.124.012836 | Methods; Figures 1-2; Table 2. |
| 5 | Knops et al. 2024 (quality of life) | 10.1161/CIRCOUTCOMES.124.010822 | Questionnaire flow; Figures 1-2. |
| 6 | Olde Nordkamp et al. 2025 (XL) | 10.1161/CIRCULATIONAHA.125.074576 | Methods; Results; Figure 1; supplementary cohort tables. |
| 7 | Healey et al. 2022 (ATLAS) | 10.7326/M22-1566 | Figure 1; Tables 2-3; study protocol and supplement. |
| 8 | Carroll et al. 2024 | 10.1093/eurjcn/zvae067 | Methods; Results; Tables 1-4. |
| 9 | Leong et al. 2024 | 10.1016/j.jacep.2024.04.034 | Figure 1; Table 2; evaluable echocardiographic samples. |
| 10 | Benz et al. 2026 | 10.1016/j.jacc.2025.10.020 | Methods; Figure 1; Table 1; linked trial populations. |
| 11 | Lee et al. 2025 | 10.1016/j.jfma.2025.06.043 | Methods; Figure 1; Tables 1-3. |
| 12 | Palmisano et al. 2021 | 10.1111/jce.15080 | Methods; matched-cohort Results; Tables 1-2. |
| 13 | Brouwer et al. 2018 | 10.1093/europace/euy083 | Data sources; Figure 1; Table 2; matched/stratified analyses. |
| 14 | Honarbakhsh et al. 2017 | 10.1016/j.ijcard.2016.11.017 | Methods; Results; Tables 2-4. |
| 15 | Liang et al. 2019 | 10.1097/CM9.0000000000000133 | Study patients and design; Table 1; component outcomes. |
| 16 | Brouwer et al. 2016 | 10.1016/j.jacc.2016.08.044 | Study setting; Table 1; clinical outcomes. |
| 17 | Viani et al. 2019 | 10.1016/j.hrthm.2018.10.026 | Study design; post-extraction population; registry identity. |
| 18 | Boveda et al. 2018 | 10.1016/j.ijcard.2017.11.104 | DOI-matched accepted manuscript; Methods and Results. |
| 19 | Mithani et al. 2018 | 10.1093/europace/eux026 | Data source; matching; Tables 1-2. |
| 20 | Al-Ghamdi et al. 2017 | 10.14740/cr638w | Study population; Results; Table 1. |
| 21 | Köbe et al. 2017 | 10.1007/s00392-016-1055-0 | Methods; responder Results; Table 1. |
| 22 | Friedman et al. 2016 | 10.1001/jamacardio.2016.2782 | Study population; three-way matching; Tables 3-4. |
| 23 | Köbe et al. 2013 | 10.1016/j.hrthm.2012.09.126 | Abstract; follow-up methods; Tables 1-2. |

**Supplementary Figure Legends**

**Supplementary Figure S1. First appropriate shock with S-ICD versus TV-ICD.** Forest plot of time to first appropriate shock. Randomised evidence from PRAETORIAN and ATLAS is primary and was synthesised using published Cox hazard ratios with a REML random-effects model. Matched observational estimates from Brouwer 2018 and Lee 2025 are shown separately as supportive evidence. The study-design interaction is exploratory and uses modified Hartung-Knapp inference. First appropriate shock does not measure total shock burden or episode-level shock efficacy. CI, confidence interval; HR, hazard ratio; REML, restricted maximum likelihood.

**Supplementary Figure S2. Device-related infection with S-ICD versus TV-ICD.** Randomised infection endpoints are shown separately because definitions and follow-up differ. ATLAS reports infection requiring surgery at 6 months, while PRAETORIAN reports infection requiring extraction and systemic device-related infection. Absolute differences per 1,000 are descriptive. The matched-cohort Mantel-Haenszel risk ratio is supportive and was calculated without a continuity correction; alternative sparse-event models are reported in Supplementary Table S2. RR, risk ratio.

**Supplementary Figure S3. Pneumothorax with S-ICD versus TV-ICD.** Source-specific randomised and matched observational pneumothorax comparisons are shown without a continuity correction. Because events were sparse and S-ICD arms contained zero events, no pooled risk ratio was forced. Exact conditional stratified tests assess association within the randomised and matched observational strata but do not provide a pooled relative-effect confidence interval. Absolute differences per 1,000 are descriptive. RR, risk ratio.

**Supplementary Figure S4. Isolated cardiac tamponade with S-ICD versus TV-ICD.** Source-specific isolated tamponade comparisons are shown without pooling. ATLAS contributes a double-zero comparison, while PRAETORIAN reports two events in the TV-ICD group and none in the S-ICD group. Matched observational results are supportive. The broader ATLAS pericardial or perforation composite was not substituted for the isolated tamponade endpoint. Absolute differences per 1,000 are descriptive.

**Supplementary Figure S5. Pacing, upgrades and device changes with S-ICD versus TV-ICD.** Source-specific parent-trial and long-term PRAETORIAN-XL data are presented descriptively because endpoints and horizons are not interchangeable. PRAETORIAN-XL is extended follow-up of the PRAETORIAN cohort and is not an independent trial. Device changes attributed to subsequent bradycardia-pacing or antitachycardia-pacing indications reflect differences in device architecture and should not be interpreted as direct comparisons of the biological incidence of new pacing indications. ATP, antitachycardia pacing; CRT-D, cardiac resynchronisation therapy defibrillator; HF, heart failure; HR, hazard ratio.

**Supplementary Figure S6. Quality of life with S-ICD versus TV-ICD.** Patient-reported outcomes from ATLAS and PRAETORIAN are shown separately because the studies used different instruments, assessment times and analytical approaches. ATLAS reports adjusted 6-month FPAS and SF-36 component-score comparisons; the FPAS analysis adjusts for the 1-month postimplant score rather than a preimplant baseline. PRAETORIAN reports 30-month DASI and SF-36 domains. No pooled standardised mean difference was calculated. DASI, Duke Activity Status Index; FPAS, Florida Patient Acceptance Survey; SF-36, 36-Item Short Form Health Survey.

**Supplementary Figure S7. Result-specific RoB 2 assessments for randomised evidence.** Traffic-light plot of result-specific risk-of-bias judgements using RoB 2. Domains are D1, bias arising from the randomisation process; D2, bias due to deviations from intended interventions; D3, bias due to missing outcome data; D4, bias in measurement of the outcome; and D5, bias in selection of the reported result. Judgements are low risk, some concerns or high risk. PRAETORIAN-XL is assessed as extended follow-up of the parent randomised cohort.

**Supplementary Figure S8. Summary of RoB 2 judgements across randomised results.** Distribution of result-specific RoB 2 judgements across the five domains and overall risk of bias. Percentages summarise assessed results rather than unique trials, because multiple outcomes were assessed separately within the same parent trial. Judgements are low risk, some concerns or high risk.

**Supplementary Figure S9. Result-specific ROBINS-I assessments for non-randomised evidence.** Traffic-light plot of result-specific ROBINS-I judgements for non-randomised comparisons. Domains are D1, bias due to confounding; D2, bias in selection of participants; D3, bias in classification of interventions; D4, bias due to deviations from intended interventions; D5, bias due to missing data; D6, bias in measurement of outcomes; and D7, bias in selection of the reported result. Judgements are low, moderate, serious or no information. The target comparison was initial S-ICD versus TV-ICD strategy with usual subsequent care.

**Supplementary Figure S10. Summary of ROBINS-I judgements across non-randomised results.** Distribution of result-specific ROBINS-I judgements across the seven domains and overall risk of bias. Percentages summarise assessed results rather than unique studies, because multiple outcomes and, where relevant, alternative analysis populations were assessed separately. Judgements are low, moderate, serious or no information.
